# Novel Strategies for Soil-Borne Diseases: Exploiting the Microbiome and Volatile-Based Mechanisms Toward Controlling *Meloidogyne*-Based Disease Complexes

**DOI:** 10.3389/fmicb.2019.01296

**Published:** 2019-06-07

**Authors:** Adrian Wolfgang, Julian Taffner, Rafaela Araújo Guimarães, Danny Coyne, Gabriele Berg

**Affiliations:** ^1^Institute of Environmental Biotechnology, Graz University of Technology, Graz, Austria; ^2^Department of Phytopathology, Universidade Federal de Lavras, Lavras, Brazil; ^3^International Institute of Tropical Agriculture, Nairobi, Kenya

**Keywords:** root-knot nematodes, tomato microbiome, biocontrol, antagonists, *Pseudomonas*, *Comamonas*, *Variovorax*, *Bacillus*

## Abstract

Under more intensified cropping conditions agriculture will face increasing incidences of soil-borne plant pests and pathogens, leading to increasingly higher yield losses world-wide. Soil-borne disease complexes, in particular, are especially difficult to control. In order to better understand soil-borne *Meloidogyne*-based disease complexes, we studied the volatile-based control mechanism of associated bacteria as well as the rhizospheric microbiome on Ugandan tomato plants presenting different levels of root-galling damage, using a multiphasic approach. The experimental design was based on representative samplings of healthy and infected tomato plants from two field locations in Uganda, to establish species collections and DNA libraries. Root galling symptoms on tomato resulted from a multispecies infection of root-knot nematodes (*Meloidogyne* spp.). Results revealed that 16.5% of the bacterial strain collection produced nematicidal volatile organic compounds (nVOC) active against *Meloidogyne*. Using SPME GC-MS, diverse VOC were identified, including sulfuric compounds, alkenes and one pyrazine. Around 28% of the bacterial strains were also antagonistic toward at least one fungal pathogen of the disease complex. However, antagonistic interactions appear highly specific. Nematicidal antagonists included *Pseudomonas*, *Comamonas*, and *Variovorax* and fungicidal antagonists belonged to *Bacillus*, which interestingly, were primarily recovered from healthy roots, while nematode antagonists were prominent in the rhizosphere and roots of diseased roots. In summary, all antagonists comprised up to 6.4% of the tomato root microbiota. In general, the microbiota of healthy and diseased root endospheres differed significantly in alpha and quantitative beta diversity indices. Bacteria-derived volatiles appear to provide a remarkable, yet wholly unexploited, potential to control *Meloidogyne*-based soil-borne disease complexes. The highly specific observed antagonism indicates that a combination of volatiles or VOC-producing bacteria are necessary to counter the range of pathogens involved in such complexes.

## Introduction

Agriculture causes long-lasting anthropogenic environmental impacts as it replaces natural vegetation, alters biogeochemical cycles, and decreases biodiversity; this defined a new human-dominated geological epoch, the Anthropocene. The soil disturbance by the conversion of land to agriculture has resulted in species extinctions 100–1,000 times higher than natural rates, and likely constitutes the beginning of the sixth mass extinction in Earth’s history (Lewis and Maslin, 2015). However, much less is known about the loss of microbial diversity (Berg et al., 2017) due to human activity than the loss of macroscopic diversity. In particular, bacteria occupy important niches and roles, linking plant microbial diversity and ecosystem functioning, such as productivity, host plant fitness and resilience (Bais et al., 2004; Tilman et al., 2012; Vandenkoornhuyse et al., 2015; Laforest-Lapointe et al., 2017). Among the most striking direct consequences of agricultural intensification is the elevated presence and impact of soil-borne pests and pathogens (Mendes et al., 2012). Soil-borne diseases are often “microbiome diseases”; they signify the result of a loss of microbial diversity and dysbiosis in soil and consequently in the rhizosphere and endosphere of plants (van Elsas et al., 2012). Once established, bacteria, fungi, and nematode pathogens accumulate, often as a synergistic combination, leading to high yield losses, which prove difficult to control (Oerke, 2006; Bennett et al., 2012). The soil environment is a complex arena, the biological permutations of which are little understood (Bais et al., 2004), especially in areas such as Africa, where assessments and understanding of the microflora and microbiota remain negligible.

Root-knot nematodes Göldi 1892 (RKN, genus *Meloidogyne*) infect over 5500 host plants, including plant species from nearly every extant known plant family (Trudgill and Blok, 2001). Species of *Meloidogyne* can be extremely polyphagous, are mainly parthenogenetic, and are highly adapted obligate sedentary plant parasites. They are regarded as the most economically important plant-pathogenic nematode group worldwide (Jones et al., 2013) and in the tropics, are viewed as the most significant biotic threat to crop production (Karssen et al., 2013; Coyne et al., 2018). Host roots are infected by freshly hatched, motile second stage juveniles (J2), which, upon establishing a feeding site behind the root tip, become sedentary, feeding from cells which it modifies to provide a constant supply of nutrients. Infection by RKN often leads to typical symptoms of root damage and gall formation, with above-ground symptoms of stunting, wilting, leaf chlorosis, reduced yield, which are symptomatic of water or nutrient scarcity (Karssen et al., 2013). Nematode-infected plants tend to be more susceptible to other diseases (Coyne et al., 2018). These unspecific above-ground symptoms, however, lead to an excess overuse of fertilizers and ineffective treatment with pesticides (Karungi et al., 2011). Excessive and frequent pesticide applications, in combination with inappropriate handling, increase the risks to human health as well as to water resources and the ecological system (Coyne et al., 2018). The impact of RKN on the host is significantly exaggerated through secondary pathogen infections, such as root rot pathogens, bacterial and fungal wilts, e.g., *Fusarium oxysporum* and *Verticillium dahliae* (Back et al., 2002; Karssen et al., 2013). These plant pathogens do not necessarily need the presence of RKN to successfully infect their hosts, but since RKN may act as casual agents, the disease can be seen as *Meloidogyne*-based disease complex. The interactions between RKN, secondary pathogens, host plant and plant-associated microorganisms lead to the resulting effects on plant health (Karssen et al., 2013). The management of RKN would therefore benefit from a more holistic approach, taking into consideration the management of soil-borne microbial complexes. A deep understanding of the plant-associated microbiomes would be beneficial, given that a proportion of microorganisms are antagonistic toward soil-borne pests and pathogens (Berg et al., 2002). One recently discovered indirect mode of plant disease prevention in bacteria is the production of volatile organic compounds (VOC) (Effmert et al., 2012; Cernava et al., 2015). VOC are semiochemicals that act as “long-range” allelochemicals in soil, which can have growth-promoting or -inhibiting effects on other microorganisms (Effmert et al., 2012) and plants (Ryu et al., 2003). The fact that VOC can have communicational, controlling or inhibitory effects that act inter- and/or intra-specifically, make them a highly interesting field of study for biological control (Effmert et al., 2012; Torto et al., 2018). However, despite their potential, VOC have received only limited attention and are yet to be fully exploited for biocontrol strategies. Our hypothesis was that RKN can be negatively affected and controlled by bacteria-derived VOC in the microbiome.

We selected tomato as a model plant, given its susceptibility to RKN and soil-borne disease complexes, using two RKN-infected field sites in Uganda. Tomatoes in Uganda are a key source of income and food security for smallholders (Ssekyewa, 2006) who often own less than 2 ha of land (Karungi et al., 2011). A major challenge in controlling the RKN-disease complex is the need for simultaneous control of all involved pathogens. Novel mechanisms are urgently needed to address these soil-borne challenges, especially in Africa where the need to sustainably intensify cropping production systems is critical (Bennett et al., 2012; Vanlauwe et al., 2014). Our current study focused on three main objectives: (i) identify the *Meloidogyne* species present, (ii) screen bacterial strains capable of producing nematicidal VOC or fungicidal metabolites, (iii) analyze the microbiome shift in the root endosphere due to RKN activity. Results of this study will create a better understanding of the soil-pathogen-host interactions in the RKN-disease complex, which will be translated to developing novel control strategies.

## Materials and Methods

### Sampling Design

Three bulk samples, each comprising ten roots of fruit-bearing tomato plants (*Solanum lycopersicum* L.) with adhering soil, were collected from two field sites in Uganda in April 2017. Gall formation was categorized according to a root galling index (RGI) from 1 (no visible galling damage) to 5 (severe/lethal damage) (Coyne et al., 2007); two plants were selected for each RGI score from each site during uprooting. Sampling site “Luwero” (0°39′20″ N, 32°24′38″ E, 1187 m) was a rural farmer’s open field with 1-year-old virgin soil. Tomato cv. “Rio Grande,” received unknown application levels of pesticides but included generic fungicides, mainly mancozeb-based and the insecticides cypermethrin and chlorpyriphos. Sampling site “Namulonge” was at the IITA research station (0°31′46″ N, 32°36′45″ E, 1170 m) and consisted of a RKN-infected sandy soil within a concrete tomato outdoor bed with no direct connection to surrounding soils. Tomato cv. “Moneymaker” received no pesticide applications. A 300 g soil sample from each sample site was assessed for pH, nutrient (K, P, Mg, organic matter) content and soil type by “AGROLAB Agrar und Umwelt GmbH” (Sarstedt, Germany) to compare soil composition between the two sampling sites.

### Bacterial Strains and Isolation of Total Community DNA

Samples were recovered from different microhabitats associated with RKN infection: bulk soil constituting the native bacterial community in the field, rhizosphere representing root-associated bacteria, galled and non-galled rhizoendosphere. Bacterial suspensions were recovered using 0.9% NaCl from a 5 g sub-sample of the bulk soil (soil between tomato plants, three samples/site, *n* = 6), from rhizosphere (root adhering soil, one/plant, *n* = 20) and surface sterilized sections of roots from both galled (RE-D, *n* = 16) and non-galled (RE-H, *n* = 17) roots. Suspensions were used for DNA extractions for both amplicon analysis and isolation of bacterial strains (for details, see Supplementary Material – Additional Methods). Suspensions were plated onto NA plates (nutrient terestingly agar; Sifin GmbH, Berlin, Germany); in total 260 strains were isolated and screened for nematicidal (see section “Screening for nVOC-Producing Strains”) and fungicidal properties (see section “Bacterial Antagonistic Activity Against Fungal Pathogens”). Extraction of the DNA pellet was conducted using “FastDNA Spin Kit for soil” (MP Biomedical, Eschwege, GER). PCR-products were cleaned with GENECLEAN Turbo^TM^ Kit (MP Biomedicals, Eschwege, GER) following the manufacturer’s instructions for genomic DNA. 16S rRNA gene amplifications were carried out in 3 × 30 μl reactions with the Illumina barcode universal bacterial primer set 515f-806r (Caporaso et al., 2011) and PNA Mix (Lundberg et al., 2013) to remove plastid DNA. PCR products of barcoded samples were pooled to equimolarity; sequencing was carried out by Eurofins MWG Operon (Ebersberg, GER^1^) with an Illumina HiSeq 2500 system (for details, see Supplementary Material – Additional Methods).

### Identification of Nematodes and Inocula Production

Randomly selected females with eggs were dissected from diseased roots. Perineal patterns of females were used for morphological diagnosis; body content of the same crushed females were used for molecular identification using the molecular key method of Adam et al. (2007). Furthermore, the region for NAD dehydrogenase subunit 5 was amplified for genetic determination using the body content of individual females (Janssen et al., 2016). Data were combined to determine species identification. Eggs were used to re-infect tomato seedlings in the fourth-true-leaf stadium to establish pure cultures. J2 of identified pure cultures were partially used in further experiments. For extracting J2, roots were rinsed free of adhering soil. Diseased root sections were chopped coarsely, placed in 1.2% NaOCl solution and blended with a hand blender for 3 min. The suspension was rinsed with tap water on nested 100–25 μm sieves. Eggs were caught on 25 μm sieve and collected into a beaker, which was aerated for 10 days to allow hatching. The J2 suspensions were placed on a Baermann funnel filter for 24 h at room temperature (Supplementary Figure S1A). The resulting ∼30 ml J2 suspension was stored horizontally in 50 ml Sarstedt tubes at 6°C until use.

### Screening for nVOC-Producing Strains

We used a variation of the two clamp VOC assay (TCVA, Cernava et al., 2015): Bacterial strains were streaked on 12-well plates containing NA and incubated at 30°C for 24 h. Each plate had a blank well, containing NA only. Plates were inversed onto another 12-well plate containing ∼100 J2 of *M. incognita* (provided from Julius Kühn-Institut, Münster, Germany) on 2%-tap water agar. A silicon foil with a 5 mm hole between the two opposing chambers separated the two 12-well plates. The two plates were clamped together to provide airtight test conditions (Supplementary Figure S1B) and then maintained for 24 h at room temperature. Dead J2 were assigned dead if the body was straight and did not react when prodded with a dissection needle. Percentage J2 mortality was calculated after correcting for the blank value of the corresponding plate blank. Bacterial strains were categorized according to their activity: non-active (<10% mortality), slightly active (>10–80%), active (>80–95%), and highly active (>95%). Distributions of the number of bacterial strains within the categories were compared between sampling sites, microhabitat (healthy/diseased root, rhizosphere) and RGI. The experiment was repeated two more times for those samples demonstrating >80% nematicidal activity. The strains showing consistent nematicidal activity were identified by sequencing of 16S rRNA gene.

### Bacterial Antagonistic Activity Against Fungal Pathogens

All 260 isolated bacterial strains were tested for their antagonistic activity against the fungal pathogens *Botrytis cinerea*, *F. oxysporum*, *Fusarium verticillioides*, *Sclerotium rolfsii*, and *V. dahliae* (provided by Institute of Environmental Biotechnology Graz) in dual cultures on Waxman agar in three replicates. Antifungal activity was categorized according to: 0 (fungi overgrow bacterial colony), +1 (hyphae reach bacteria, but do not overgrow), +2 (lateral inhibition zone <0.5 cm), and +3 (lateral inhibition zone >0.5 cm). The mean category across the three repeat assessments was calculated. Bacterial strains showing a strong antifungal effect (category +3) were compared using VENN^2^. DNA of strains with a mean antifungal activity of +3 against at least four pathogens (*n* = 23, see also Figure 2A) was extracted. A BOX-PCR was carried out to identify clones, resulting in five genotypes. 16S rRNA gene of the resulting five genotypes was amplified and sequenced for identification.

### SPME GC-MS of nVOC and Nematicidal Effect of Single Compounds

Nematicidal volatile organic compounds were identified using an adapted version of the method from Verginer et al. (2010) (for details, see Supplementary Material – Additional Methods). A total of nine compounds (purity >98%) partially found in the GC-MS samples were tested against *M. javanica* J2 in a chambered Petri dish, namely decene (10en), undecene (11en), undecane-2-on (11on), dodecene (12en), 2-methoxy-3-methyl pyrazine (2M3MP), 2,5-dimethyl pyrazine (25DP), 5-isobutyl-2,3-dimethyl pyrazine (5I23DP), 2-ethyl-3-methyl pyrazine (2E3MP), and 2-isobutyl-3-methoxy pyrazine (2I3MP) (all Sigma-Aldrich, Darmstadt, Germany). 2M3MP was used as a substitute for 3-methoxy-2,5-dimethyl pyrazine, which was consistently detected in nVOC-volatilome of *Pseudomonas koreensis* T3GI1 (see also Table 2). The compound 2-undecanone was used as a positive control, due to its known nematicidal effects on *M. incognita* (Huang et al., 2010) and other nematodes (Gu et al., 2007). On one side of the Petri dish, a 500 μl suspension of *M. javanica* (∼250 J2) was placed on 8 ml of 2%-tap water agar. On the opposite side, three concentrations (1, 5, and 20 μl) of a single compound were placed on a microscopic slide, which prevented interactions of the compound with the Petri dish plastic. A Petri dish with 20 μl distilled water represented the control. Plates were maintained at room temperature for 24 h and the experiment repeated a further two times and the blank-corrected mortality rate calculated for each compound.

### Amplicon Analysis and Statistics

Pre-processing of the reads obtained by the sequencing company of Eurofins MWG Operon was carried out using QIIME 2 (2017.12 release) and QIIME 1 (Caporaso et al., 2010) following the protocol of Schwendner et al. (2017). Demultiplexing, denoising (400 bp length, including phiX and chimera filtering) and generation of ribosomal sequence variants (RSVs) was carried out with the DADA2 algorithm (Callahan et al., 2016). RSVs were summarized in a feature table. The taxonomic analysis was based on a customized naïve-bayes classifier trained on 16S rRNA gene features clustered at 97% similarities within the Silva128 database release and trimmed to a length of 400 bp. Reads for mitochondria and chloroplasts were filtered using QIIME 2 before analysis. The feature table was rarefied to 6,890 reads for core metrics analysis. Alpha diversity indices were analyzed using Pairwise Kruskal–Wallis test and beta diversity indices with PERMANOVA. Bray–Curtis dissimilarity and unweighted UniFrac dissimilarity between habitats were visualized using EMPEROR^3^. One rhizosphere sample (T4R) was removed due to poor quality. Differential abundances were subjected to ANCOM and Gneiss test, implemented in QIIME 2. Bacterial network of the core microbiome of each habitat (>1% relative abundance within habitat) was visualized using Cytoscape 3.3.0 (Shannon et al., 2003) on order level. The core microbiomes were defined with an occurence of >75% throughout the replicates for each habitat. Mean relative abundance of fungal and nematode antagonists found in this study was calculated for each data set. Bacterial abundances on family level were compared between healthy and diseased root samples to visualize bacterial community shift due to RKN infection.

## Results

### *Meloidogyne* Were the Causal Agents of the Disease Complex in Tomato

The combination of identification approaches (SCAR-primer molecular key, morphological examination, NAD5 sequences) identified 10 adult females as *M. incognita* and two as *M. incognita* sensu lato. in Luwero (site 1). Just *M. incognita* was reliably identified from Luwero. In samples from Namulonge (site 2), three females were identified as *M. incognita*, six as *M. javanica* and five as *M. incognita* s. lat. Thus, a multispecies infection was confirmed in Namulonge (see Supplementary Material – Additional Results, Supplementary Figures S2, S3).

### Bacterial Strains With Antagonistic Activity Toward *Meloidogyne*

**FIGURE 1:**
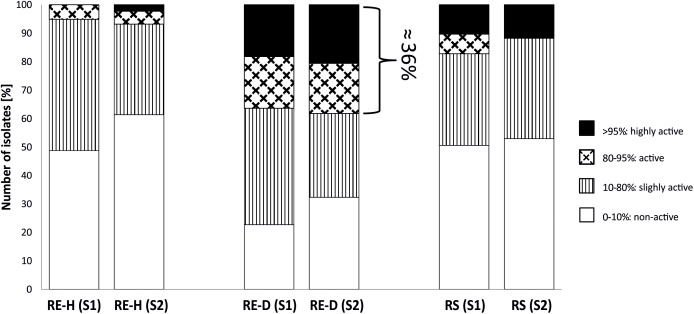
Proportion of nematicidal active bacterial strains. Proportion of nematicidal active strains is highest in diseased root endosphere, independent of site. RE-H, rhizoendosphere healthy; RE-D, rhizoendosphere Meloidogyne-diseased; R, rhizosphere; S1, Site 1, Luwero; S2, Site 2, Namulonge; Pattern refer to the activity category; in RE-D, around 36% of bacterial strains are categorized active or very active (>80% mortality) in nVOC-production.

Representative bacterial strains from each of the two sampling sites caused similar mean nematode mortality rates through nVOC (32.1 ± 25.4% in Luwero, 30.9 ± 28.1% in Namulonge). Most strains showed no effect (mortality <10%) or only slight nematicidal effects. In total, 43 strains were categorized as active (80–95% mortality; *n* = 20) or highly active (>95% mortality; *n* = 23). When comparing the overall nematicidal activity of bacteria from plants of different RGI, a trend was apparent for higher mortality rates of strains from highly diseased plants. Most active and highly active strains (*n* = 43) were recovered from diseased rhizoendosphere (RE-D) samples, independent of site (Figure 1). Of these 43 strains, six demonstrated repeated nematicidal activity (>80% mean mortality). Five out of these six highly active strains were bacteria isolated from RE-D. Moreover, three of these strains were collected from the same tomato plant (T13), a heavily galled (RGI = 4.5) plant from Namulonge. Just one strain was isolated from healthy, non-galled rhizoendosphere (RE-H) from Luwero. Sequences of the 16S rRNA genes identified the nematicidal bacteria as *Pseudomonas koreensis*, *Comamonas sediminis*, *Variovorax paradoxus*, *P. soli* and two strains of *P. monteilii*. Strains with high nematicidal activity showed no high antifungal activity and *vice versa* (Table 1).

**Table 1:** Identification of bacterial strains with antagonistic properties against either root-knot nematodes or fungal pathogens.

Strain ID	Best hit (ref_seq)	Ident.	NCBI Acc. No.	Identified as	Mortality	Antifungal category				
						*B. c.*	*F. o.*	*F. v.*	*S. r.*	*V. d.*
T3GI1	*Pseudomonas koreensis*	99%	NR_025228.1	*P. koreensis* T3GI1	97.84%	1	2	2	0	1
T1GI1	*Variovorax paradoxus*	99%	NR_113736.1	*V. paradoxus* T1GI1	96.19%	0	1	1	0	1
T13GI2	*Comamonas sediminis*	99%	NR_149789.1	*C. sediminis* T13GI2	93.77%	1	1	2	1	0
T8GH4	*P. monteilii*	100%	NR_114224.1	*P. monteilii* T8GH4	87.66%	0	1	0	0	0
T13GI4	*P. soli*	100%	NR_134794.1	*P. soli* T13GI4	86.68%	2	1	2	2	2
T13GI6b	*P. monteilii*	100%	NR_114224.1	*P. monteilii* T13GI6b	83.54%	0	1	2	0	1
T3R11	*Bacillus velezensis*	98%	NR_075005.2	*B.* cf. *velezensis* T3R11	1.9%	3	3	3	3	1
T17GI1	*Bacillus velezensis*	97%	NR_075005.2	*B.* cf. *velezensis* T17GI1	36.56%	3	3	3	3	1
T17GI2	*B. amyloliquefaciens*	99%	NR_117946.1	*B. amyloliquefaciens* T17GI2	3.47%	3	3	3	3	2
T7GH4a	*B. methylotrophicus*	99%	NR_116240.1	*B. methylotrophicus* T7GH4a	0%	3	3	3	3	0
T21GH5	*B. velezensis*	97%	NR_075005.2	*B.* cf. *velezensis* T17GI1	0%	3	3	3	3	1
T1R1	–	–	–	T1R1	6.52%	2	2	2	2	3
T1R12	–	–	–	T1R12	4.76%	2	2	2	0	3
T1R14	–	–	–	T1R14	4.11%	2	2	2	2	3
T2R18b	–	–	–	T2R18b	0%	2	2	2	2	3
T14R6	–	–	–	T14R6	3.07	2	2	2	1	3


*Mortality: mean mortality of *Meloidogyne incognita* juveniles due to bacterial volatiles. Antifungal category: mean values for antagonism against *Botrytis cinerea* (*B. c*.), *Fusarium oxysporum* (*F. o*.), *F. verticillioides* (*F. v*.), *Sclerotium rolfsii* (*S. r*.), and *Verticillium dahliae* (*V. d*.), where 0, no antagonism; 1, hyphae do not overgrow colony; 2, inhibition zone <5 mm; and 3 = inhibition zone >5 mm. Values for nematode antagonists and fungal antagonists with five examples of *V. dahliae*-antagonistic strains.*

The volatilomes of the nVOC-producing strains mainly consisted of alkenes, sulfuric compounds, alcohols, ketones, and aldehydes. Additionally, one pyrazine (3-methoxy-2,5-dimethyl pyrazine) was consistently detected in *P. koreensis* T3GI1, the strain with the highest nematicidal effect (Table 2). 1-undecene was one of the main components of the volatilome of *C. sediminis*, *P. monteilii*, and *P. soli*. Except for *P. monteilii* T8GH4, dimethyl disulfide was the main component of the volatilome but strangely, was also found in the blank (Table 2). When comparing the overall number of mass spectra ion counts, the total ionic counts of bacterial volatilomes were up to sevenfold higher (in *P. soli* T13GI4) than in the blank (Table 2). When individually assessing 2-undecanone, several alkenes and pyrazine derivates against J2, just three compounds, 2-undecanone (11on), 2-methoxy-3-methyl pyrazine (2m3mp), and 2-ethyl-3-methyl pyrazine (2e3mp), gave significantly higher mortality rates than the blank mortality after 1-day incubation using quantities of 5 and 20 μl. Only 11on and 2m3mp gave significantly higher mortality rates than the blank when using 1 μl of compound (Supplementary Figure S4). At the highest concentration, live J2 were mainly found surrounded by or within a mass of dead J2 individuals. Three more compounds showed nematicidal effects at 20 μl following incubation for 4 days, namely 2-isobutyl-3-methyl pyrazine (67.8%), 5-isobutyl-2,3-dimethyl pyrazine (44%), and 2-ethyl-3-methyl pyrazine (50.2%).

**Table 2:** SPME GC-MS: volatilome composition of nematicidal VOC-producing bacterial strains associated with root-knot nematode infection in Ugandan tomato.

Compounds	RI measured	RI NIST (mainlib)	NIST match	*P. koreensis* T3GI1	*V. paradoxus* T1GI1	*C. sediminis* T13GI2	*P. monteilii* T8GH4	*P. soli* T13GI4	*P. monteilii* T13GI6b	blank (NA)
**Alkenes**										
1-nonene	889	892	868	–	–	0.68	1.96	–	–	–
1-decene	989	993	933	–	–	0.78	0.94	–	–	–
1-undecene	1091	1093	932	1.2	–	47.12	68.43	17.83	28.45	–
E-1,4-undecadiene	1081	–	–	–	–	–	0.99	–	–	–
E-3-undecene	1085	1085	884	–	0.3	–	–	–	–	–
1-dodecene	1186	1193	913	–	–	0.38	0.6	–	–	–
1-tridecene	1286	1293	919	–	–	–	0.04	–	–	–
1,12-tridecadiene	1272	1279	871	–	–	–	0.08	–	–	–
**Sulfuric compounds**										
Methanethiol	–	464	976	4.45	5.39	2.03	1.03	2.27	1.13	–
Dimethyl sulfide	520	515	745	1.73	–	0.55	–	1.01	1.64	–
Dimethyl disulfide	733	740	968	70.43	81.69	44.44	14.19	73.6	39.01	25.38
Dimethyl trisulfide	959	972	926	0.62	2.88	–	–	–	6.58^a^	16.37
S-methyl propanethioate	784	785	781	–	1.44	–	2.25	–	–	–
2,3-dimercaptopropan-1-ol	835	–	680	1	–	–	–	–	–	–
2-me-2-methylthiobutan	836	847	755	–	–	–	0.17	–	–	–
Methyl thiolacetate	698	701	877	–	1.2	–	5.45	–	–	–
S-methyl 3-methyl-butanethioate	931	938	727	–	–	–	0.13	–	–	–
S-methyl ester octanethioic acid	1291	1293	809	–	–	–	0.16	–	–	–
**Oxygen-containing compounds**										
CO_2_	–	–	999	2.92	2.66	1.83	1.43	2.54	3	–
Acetone	502	503	741	0.84	1.7	1	0.5	1.09	1.32	0.79
2-butanone	601	601	820	1.56	1.5	1.19	0.29	1.25	3.31	3.89
2-methyl butanal	656	659	838	–	–	–	–	–	2.03	7.82
3-methyl butanal	646	649	949	–	–	–	–	–	3.6	13.82
2-methyl butanol	728	736	739	–	0.27	–	–	–	–	–
3-methyl butanol	725	734	799	–	0.96	–	0.25	–	–	–
2,3-epoxybutane	510		602	–	–	–	–	0.17^a^	–	–
Benzaldehyde	951	958		–	–	–	–	–	9.93^a^	31.93
2-undecanone	1288	1291	949	–	–	–	1.11	–	–	–
3-methoxy-2,5-dimethyl pyrazine	1046	1054		14.95	–	–	–	–	–	–
**Others**										
Methyl (Z)-N-hydroxybenzene-carboximidate	899		813	0.15	–	–	–	–	–	–
Unidentified substance (Rt = 1.581)				0.16	–	–	–	–	–	–
Unidentified substance (Rt = 12.277)				–	–	–	–	0.24	–	–
(Total ion counts)/(total ion counts blank)				5.59	6.02	5.17	2.95	7.38	1.85	1


*Values of compounds are percentage of area of ionic counts. RI, retention index for non-isothermal GC; NA, nutrition broth agar. For identification references, see Supplementary Table S1; for mass spectra, see Supplementary Table S2. ^*a*^Compound was not found in all repeats.*

### Bacterial Strains With Antagonistic Activity Toward Fungal Pathogens

**FIGURE 2:**
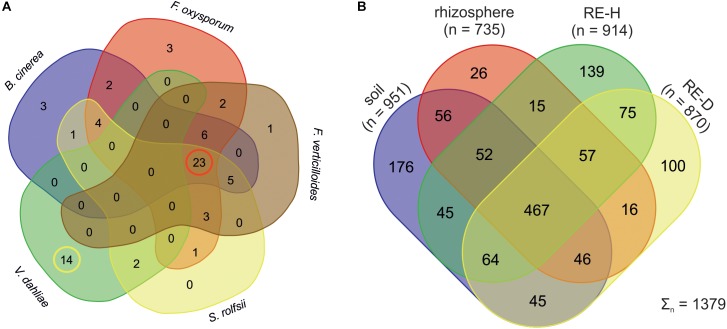
**(A)** Venn diagram of bacterial strains showing high antagonism against fungal pathogens. Strains that showed high antagonism against *Botrytis cinerea*, *Fusarium oxysporum*, *F. verticillioides*, and *Sclerotium rolfsii* did not show high effects against *Verticillium dahliae*. Red circle: active strains against four fungal pathogens. Yellow circle: *Verticillium* antagonists. **(B)** Venn Diagram displaying the number of taxa on genus level of bacterial strains. Rhizosphere sample have the least microhabitat-specific taxa. Endosphere samples have more specific than shared taxa.

Of 260 bacterial strains 72 showed a high antagonistic effect on at least one fungal pathogen tested *Botrytis cinerea*, *F. oxysporum*, *F. verticillioides*, *S. rolfsii*, and *V. dahliae* (Figure 2A). Strains showing a fungal antagonistic effect were mostly isolated from RE-H (*n* = 30 strains) rather than from RE-D (*n* = 14 strains) samples. Antagonists of *V. dahliae* were recovered only from the root adhering soil, or “rhizosphere.” Further analysis was carried out only with strains showing antagonism toward at least four different plant pathogenic fungi (*n* = 23, Figure 2A), thus *Verticillium* antagonists were excluded. Five separate genotypes were revealed by analyzing the BOX-PCR patterns of the 23 strains that complied with this requirement. Sequencing the 16S rRNA gene of these strains identified them all as members of the *Bacillus amyloliquefaciens* complex, namely one strain of *B. amyloliquefaciens* (T17GI2), one strain of *B. methylotrophicus* (T7GH4), and three strains of *B. velezensis* (T3R11, T17GI1, T21GH5). None of these strains showed high antagonistic activity against *V. dahliae* (Table 1).

### Deciphering the Tomato Microbiome and Surrounding Soil Microbiome

**FIGURE 3:**
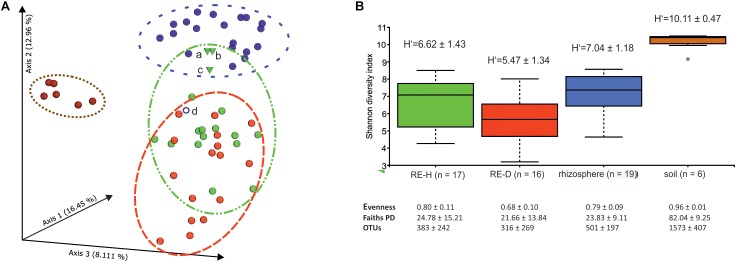
**(A)** PCoA plot of Bray–Curtis dissimilarity between microhabitats. PCoA-plot shows clear clustering in soil (brown) in rhizosphere (blue) while healthy rhizoendosphere (green) sampling points (a-c) overlap with *Meloidogyne*-diseased rhizoendosphere (red) and rhizosphere; sample point d (rhizosphere) does not cluster with other rhizosphere samples and was removed in statistical analysis. **(B)** Barplots of Shannon diversity of microhabitats. Alpha diversity is highest in soil and lowest in diseased rhizoendosphere (galls). Alpha diversity index values are given.

Physical soil composition was comparable at both sampling sites; soil type was loamy sand (Table 3). Amplicon libraries of different tomato microhabitats (rhizosphere; RE-H: rhizoendosphere healthy; RE-D: rhizoendosphere diseased) as well as from surrounding soil were analyzed. When taxa were compared at the genera level between all four microhabitats, a total of 1,379 taxa were found with 467 taxa present in all microhabitats. The rhizosphere presented the smallest number of microhabitat-specific taxa, while RE-H and RE-D had more microhabitat-specific than shared taxa (Figure 2B). When visualizing beta diversity with a PCoA-plot of Bray–Curtis dissimilarities, samples from the same sampling site did not cluster. Axis 1 (16.45% variation explained) showed no two-dimensional clustering of the samples in combination with axis 2 (12.96%) or axis 3 (8.11%), but samples clearly cluster habitat-specific with axis 2 and 3 (Supplementary Figures S5A–C). The combination of all three axes 1–3 shows a clear clustering of soil, rhizosphere and RE-D, while RE-H samples overlap with rhizosphere and RE-D (Figure 3A).

**Table 3:** Physical soil parameters at the two sampling sites.

Sampling site	Soil type	pH	K (mg/kg)	P (mg/kg)	Mg (mg/kg)	Org. matter (%)
Luwero	S’l	5.3	142	40	250	3.9
Namulonge	S’l	5.6	213	31	196	3.2


*The two soils mainly differ in potassium concentration. S’l: loamy sand.*

Shannon alpha diversity of the four microhabitats showed a high alpha diversity in soil and a decline in alpha diversity from rhizosphere to RE-H to RE-D (Figure 3B). Pairwise Kruskal–Wallis test for alpha diversity indices showed highly significant (*p* < 0.01) differences in alpha diversity between all microhabitats in most cases. Only rhizosphere and RE-H samples failed to differ significantly in all tested alpha diversity indices (Figure 3B and Table 4). Pairwise PERMANOVA test of beta diversity indices significantly differed between all microhabitats, except qualitative indices (Jaccard and unweighted UniFrac distance) in RE-H and RE-D samples (Table 4).

**Table 4:** *P*-values of diversity indices reveal significant microhabitat-specific bacterial community differences in root-knot nematode-diseased tomato roots.

	Alpha diversity indices				Beta diversity indices			
	Shannon	Observed OTUs	Faith’s PD	Evenness	Jaccard	Bray–Curtis	Unweighted Unifrac	Weighted UniFrac
RE-H/RE-D	*0.028*	0.505	0.746	***0.002***	0.061	*0.022*	0.396	*0.013*
RE-H/R	0.350	0.084	0.788	0.579	<***0.001***	<***0.001***	<***0.006***	<***0.003***
RE-H/S	<***0.001***	<***0.001***	<***0.001***	<***0.001***	<***0.001***	<***0.001***	<***0.001***	<***0.001***
RE-D/R	<***0.002***	<***0.004***	0.289	<***0.001***	<***0.001***	<***0.001***	<***0.001***	<***0.001***
RE-D/S	<***0.001***	<***0.001***	<***0.001***	<***0.001***	<***0.001***	<***0.001***	<***0.001***	<***0.001***
R/S	<***0.001***	<***0.001***	<***0.001***	<***0.001***	<***0.001***	<***0.001***	<***0.001***	<***0.001***


*P-values for Kruskal–Wallis and PERMANOVA results of alpha and beta diversity indices comparing microhabitats, significant (<0.05) *p*-values formatted italic, highly significant (*p* < 0.01) values formatted bold. RE-H, healthy rhizoendosphere; RE-D, *Meloidogyne*-diseased rhizoendosphere; R, rhizosphere; S, bulk soil.*

**FIGURE 4:**
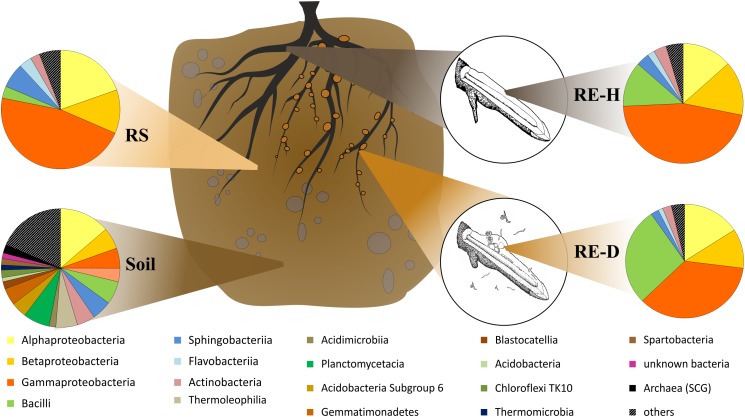
Microbial composition on class level within the four microhabitats. In diseased rhizoendosphere (RE-D), mainly Bacilli and Gammaproteobacteria change in their abundances compared to healthy rhizoendosphere (RE-D). RE-D, *Meloidogyne*-diseased rhizoendosphere; RE-H, healthy rhizoendosphere; RS, rhizosphere.

**FIGURE 5:**
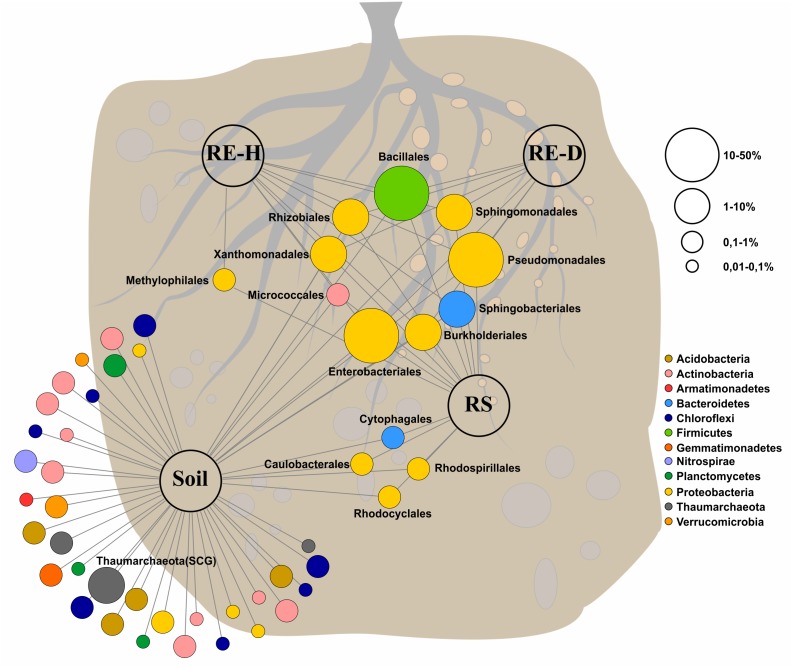
Feature network of core taxa (≥80% of samples) at class level (>1% relative abundance) found within the four microhabitats. Bacterial community in soil is very diverse, all endosphere taxa belong to the core microbiome, Methylophilales are <1% abundant in diseased endosphere. Core microbiome covers around 90% of all abundant orders except for soil (soil: 68.9%; rhizosphere: 92.7%; healthy rhizoendosphere/RE-H: 91.27%; *Meloidogyne*-diseased rhizoendosphere/RE-D: 91.24%).

Amplicon analysis of the soil samples revealed a total of 763 taxa at the genus level in Luwero (site 1) and 782 in Namulonge (site 2), with 594 taxa shared between sites. The most abundant classes in soil were Alphaproteobacteria (13.7%), Planctomycetacia (7.2%), and Bacilli (6.2%) (Figure 4). Caulobacterales, Cytophagales, Rhodocyclales, and Rhodospirillales are shared between soil and rhizosphere samples (Figure 5).

All plant-associated samples were dominated by Gammaproteobacteria. The classes most abundant in the rhizosphere were Gamma- (47.4%), Alpha- (19.8%), and Betaproteobacteria (11.6%) (Figure 5). The most abundant bacterial genera in the rhizosphere were *Pseudomonas* spp. (25.5%), unidentified Enterobacteriaceae species (17.2%) and *Sphingobium* spp. (8.2%). Endosphere samples mainly consisted of orders of the core microbiome (Figure 5).

**FIGURE 6:**
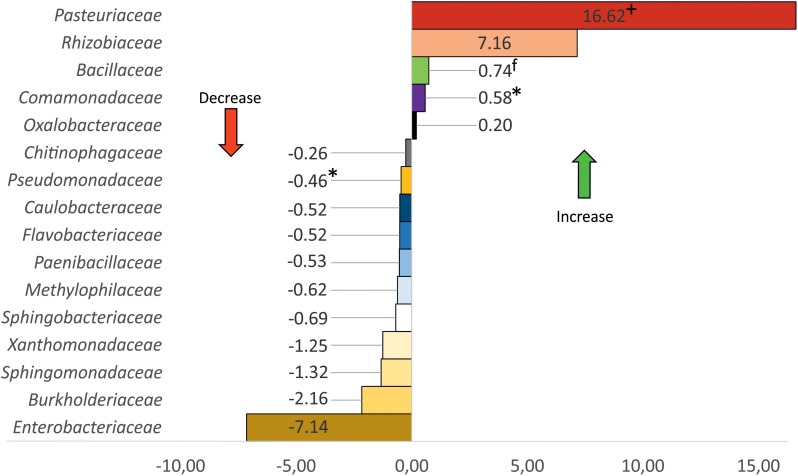
Changes of relative abundance of bacteria families due to root-knot nematode (*Meloidogyne* spp.) infection. Only families with >1% relative abundance shown. +, obligate nematode parasites; f, fungi antagonists; ^∗^, nematode antagonists found in this study.

Differential abundance analysis using Gneiss proved that the “habitat” category is the main driver of variance within our dataset, changes of the microbiome composition through higher RGI were not significant. Enterobacteriaceae, Burkholderiaceae, Pasteuriaceae, and Rhizobiaceae were identified as the main drivers of microbiome changes in rhizoendosphere (Supplementary Figures S6A–D). When comparing core taxa (present in >75% of the samples) on family level, these families showed a strong abundance shift (Figure 6).

### Linking the Microbiome With Antagonistic Strains

**FIGURE 7:**
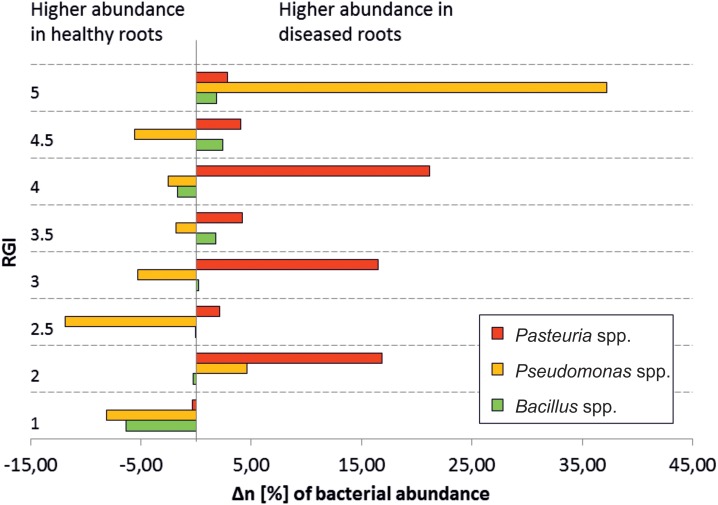
Abundance of selected nematode-antagonistic genera in relation to severity of *Meloidogyne*-infection. ΔrA, difference of relative abundances, = abundance (diseased root) – abundance (healthy root); RGI, root galling index; 1, symptomless; 5, severely damaged roots; antagonistic genera are generally higher in healthy roots, except for obligate nematode parasites *Pasteuria* spp. The high abundance of *Pseudomonas* in the RG5 plant may be due to saprophytic species, which already degrade the plant material.

Antagonists differ in their overall abundance between microhabitats. *Bacillus* spp. had the highest mean abundance in RE-H (4.1%), *Variovorax* spp. in RE-D (1%), *Comamonas* spp. (0.3%), and *Pseudomonas* spp. (22%) across the 19 rhizosphere samples. Taxa showing >98% identity with 16S rRNA sequences of isolated antagonists were represented by 6% of the root endosphere microbiome (Table 5). Focusing on the three most abundant antagonistic genera (*Pseudomonas*, *Pasteuria*, *Bacillus*), no direct correlation between their abundance and RGI was detected (Figure 7). *Pseudomonas* was especially abundant in diseased plants with a RGI of 5, whereas *Pasteuria* showed highest abundance in moderately diseased (RGI = 3 ± 1) plants (Figure 7).

**Table 5:** Relative abundance of taxa most similar to 16S rRNA sequences of isolated bacterial antagonists in different microhabitats.

Relative abundance of antagonists	Ident. (%)	n(taxa)	Soil	Rhizosphere	RE-H	RE-D
Best hit *Pseudomonas koreensis* T3GI1	98.97	7	0	0.176	0.189	0.000
*Pseudomonas putida*-group	>98	18	0.331	23.295	5.515	5.338
Best hit *P. monteilii* T8GH4	98.97		0	0.335	0.131	0.084
Best hit *P. soli* T13GI4	98.63		0.031	1.691	0.069	0
Best hit *P. monteilii* TT13GI6b	98.97		0.044	2.828	0.185	0
*Variovorax*	>98	5	0.052	0.677	0.538	1.060
Best hit *V. paradoxus* T1GI1	98.97		0.008	0.084	0.046	0
*Comamonas*	>98	4	0	0.138	0.033	0.017
Best hit *C. sediminis* T13GI2	98.97		0.025	0	0.004	0
nVOC antagonists total	>98		0.382	23.972	6.053	6.398
*Pasteuria*	>98	7	0.002	0.118	3.115	17.948
Best hit *P. penetrans* (AF077672.1)	98.97		0	0	0.136	1.104
*Bacillus amyloliquefaciens*-group	>98	6	0.008	0.141	0.286	0.128
Best hit *B. amyloliquefaciens* s. lat.	99.32		0	0	0.007	0


*n(taxa) refers to the number of taxa with >98% identic 16S rRNA sequences. RE-H, rhizoendosphere healthy; RE-D, *Meloidogyne*-diseased rhizoendosphere.*

## Discussion

### Bacteria and nVOC

In our comprehensive study of soil-borne diseases of tomato from Uganda, we discovered novel principles, which help to explain the disease complex and offer new potential strategies as to how to suppress them. Diseased tomatoes suffered from a multispecies infection of *Meloidogyne*. We clearly identified two species (*M. incognita*, *M. javanica*) but results indicate that more species are involved (Adam et al., 2007; Janssen et al., 2016).

Our novel VOC-based screening method toward RKN resulted in the identification of several nematicidal antagonists, namely *Comamonas sediminis*, *P. koreensis*, *P. monteilii*, *P. soli*, and *Variovorax paradoxus*. *P. koreensis* and *P. monteilii* have known potential for biocontrol against oomycetes (Hultberg et al., 2010a,b) and fungi in general through VOC, respectively (Dharni et al., 2014). In contrast, *P. soli* and *C. sediminis* have not previously been identified as potential biocontrol candidates. However, the closely related *C. acidovorans* has shown antagonistic effects toward plant pathogens (El-Banna, 2007; Liu et al., 2007). *Variovorax paradoxus* has great potential for bioremediation, biotechnology (Satola et al., 2013), and plant-protection (Chen et al., 2013). *Variovorax* spp. and *Comamonas* spp. occurred in relatively low abundance in endosphere samples (Table 5). The question remains as to whether they can become sufficiently abundant to affect RKN through their nematicidal nVOC and support the plant indirectly, or if they inherit direct plant-promoting traits.

A considerable aspect of the cultivable bacteria isolated in our study demonstrated their negative impact on RKN by producing nVOC. VOC produced by the bacterial community – among other factors – may contribute to the overall suppressiveness toward RKN of different soils. This may also explain the efficacy of RKN management methods that promote bacterial growth and diversity, e.g., the use of soil amendments (Viaene et al., 2013). We identified six nVOC-producing strains and 72 potential fungal antagonists. However, our tests did not result in any single strain that controlled both RKN and phytopathogenic fungi to any great extent. Furthermore, individual antagonists tended to attain high abundances in different, separate microhabitats. Fungal antagonists were mainly isolated from, and abundant in, RE-H, as supported by the amplicon data, indicating an important role of these strains for host-plant protection. The strong antagonistic effects on fungal pathogens by members of the *B. amyloliquefaciens* complex is well-known (Chowdhury et al., 2015). The strains found in our study did not effectively inhibit growth of *V. dahliae* in dual cultures, although *in vitro* antagonism toward *V. dahliae* was been reported for other *Bacillus* strains (Danielsson et al., 2007). The antimicrobial activity of their metabolites is well-studied, showing that induced systemic resistance (ISR) and antimicrobial metabolite production are their main mechanism of plant protection (Chowdhury et al., 2015). Our findings exclude nVOC of *Bacillus* strains as a controlling component for RKN, though they may have a repellent effect *in vitro*. We question that *Bacillus* spp. in the root endosphere are enriched from surrounding soil, because *Bacillus* spp. are a part of the core microbiome of the tomato seeds itself (Bergna et al., 2018). In this case, susceptibility of tomatoes toward diseases may also be a consequence of declining bacterial diversity through breeding practices or the inability to establish native antagonists.

One component of our most effective nVOC-producing strain was a pyrazine, which have known antimicrobial effects (Haidar et al., 2016; Kusstatscher et al., 2017), we therefore focused on different pyrazines in the single compound test. We found that the effect of pyrazines on RKN appears dependent on their functional groups. Although all pyrazines were not as effective as 2-undecanone, they may enhance the nematicidal effect of the total volatilome of *P. koreensis* T3GI1. Alkenes were consistently found in high concentrations in the nVOC spectra but they showed no nematicidal effect. This may be a result of their hydrophobicity, as J2 were protected by a thicker water layer in the single compound test, than in the modified TCVA-screening. Therefore, hydrophobic compounds would be less able to affect the nematode cuticle. We also found several sulfuric compounds with strong odors (e.g., dimethyl sulfide, octanethioic acid S-methylester). Nematicidal properties may mostly arise from dimethyl disulfide, which is an effective nematicide (Gómez-Tenorio et al., 2015) and was the main volatilome component in all strains except *P. monteilii* T8GH4 in our assessment. Sulfur is generally regarded to be in low concentrations in tropical soils (Blair et al., 1980). As elemental sulfur is known to reduce RKN densities in non-sterile soil (Rumiani et al., 2016), VOC of the microbial sulfur metabolism may enhance RKN control. Although our study revealed the potential of VOC in suppressing multispecies plant diseases, in order to be able to translate this concept into plant protection a greater understanding and more research is needed.

### The Microbiome of *Meloidogyne* Disease Complexes

Our study provides the first in-depth analysis of the influence of RKN on the bacterial community under field conditions. Results of Tian et al. (2015), which studied microbiomes of RKN-diseased tomatoes under controlled, greenhouse conditions differ heavily from our study in terms of the dominating taxa, microbiome composition, and microbiome shift due to RKN infection. These differences are not entirely surprising, however, given that our study was performed on plants removed from fields following natural infection by RKN, compared with the controlled pot environment and artificial inoculation of RKN by Tian et al. (2015). Some of this variance may also be due to cultivar differences (Rybakova et al., 2017) but which is not expected to be apparent at this taxonomic level. Infection with nematodes was correlated with a strong bacterial community shift in tomato roots, with a microbiome from healthy plants differing from infected roots, even though this was not necessarily dependent upon the RGI. Regarding the beta diversity, only quantitative indices revealed significant differences between RE-D and RE-H. Thus, nematode feeding site (NFS) induction would appear to have a greater impact on the abundance of bacterial taxa that are present and highly abundant in both RE-D and RE-H (Figure 6), rather than the microhabitat-specific colonization pattern of low-abundant taxa (Figure 2B). A higher diversity of endophytes due to RKN infection (Tian et al., 2015) could not be confirmed; cultivable bacteria and habitat-specific taxa were more abundant in RE-H than in RE-D. Further, RE-H and rhizosphere did not significantly differ in alpha diversity indices. This appears to be extraordinary: the rhizosphere is regarded as a biodiversity hotspot and root endosphere diversity in tomato was found to be lower than in rhizosphere. Nevertheless, both rhizosphere and to a lesser extent root endosphere diversity are influenced by the surrounding soil (Bergna et al., 2018). Since tomato are not indigenous to Uganda, the rhizoendophytic alpha diversity may be raised because of the combination of tomato core microbiota and an uptake of native soil bacteria. Interestingly, alpha diversity in RE-D is significantly lowered compared to RE-H despite comparable numbers of species. Therefore, RKN may favor roots with lower microbiome diversity for NFS selection. Diversity of endophytic microbiota is regarded as a key factor for plant health (Berg et al., 2017) but RGI and Shannon diversity do not clearly correlate with healthy and diseased roots (data not shown). Furthermore, RE-D microbiomes of moderately damaged plants (RGI 2–3.5) show a more asymmetric composition with fewer but more dominant taxa than the severely damaged plants (RGI = 4.5–5). This may be due to delayed establishment of slower growing saprophytic or commensal species, although, Tian et al. (2015) also found a specific enrichment of some bacterial groups in the NFS, which indicated specific association of these groups with the NFS and nematode pathogenesis. Our results indicate that the changed physiological conditions of plant cells at the NFS is responsible for microbiome changes. The microbial community within the NFS is influenced by microbes that are able to adhere to the nematode cuticle (Elhady et al., 2017). This is most obvious when looking at the abundance of obligate parasites of plant pathogenic nematodes, such as *Pasteuria* spp. within RE-D samples (six samples with >20% relative abundance). Their abundance did not correlate with RGI (Figure 7), maybe because it is dependent on a successful transportation adhered to the cuticle of RKN to the NFS. The increase of Rhizobiaceae in RKN galls seems to be a constant effect (Hallmann et al., 2001; Tian et al., 2015). There are three possible explanations: (i) Rhizobiaceae have a preference to attach to the nematode surface during soil migration, as reported for *Neorhizobium* (Elhady et al., 2017); (ii) it is a side effect of NFS induction, since RKN manipulate the gene expression of plant hormones and nodulation factors (Gheysen and Fenoll, 2002; Jones et al., 2013); (iii) it contributes to a defense reaction of the host plant, since Rhizobiaceae are known to closely interact with plants. The latter two hypotheses are connected to the regulation of plant flavonoids, which have several important functions in the plant-nematode interaction (Hutangura et al., 1999; Weston and Mathesius, 2013) and there is evidence that RKN counteract the effects of *Rhizobium*, since RKN reduce nodulation in legumes (Kimenju et al., 1999). Enterobacteriaceae and Burkholderiaceae were the families with the highest negative shift in abundance in RE-D. Whether there was a lower abundance at the NFS beforehand, or this was lowered following RKN infection remains unclear. However, Enterobacteriaceae and Burkholderiaceae appear to be less competitive following the physiological and physical changes that occur as galls develop in the roots. Due to the high abundance of Enterobacteriaceae (31.8%) and Burkholderiaceae (5.3%) in RE-H their abundance shift is most obvious. Still, when examining the abundance changes separately, the percentage change in abundances is higher in low-abundant taxa, such as Caulobacterales or Methylophilales (Supplementary Table S3).

Managing the RKN disease complexes through biocontrol requires a detailed knowledge on the antagonists and their effects. Since antagonistic strains of bacteria are here shown to prefer different microhabitats, they would likely affect RKN at different stages of their life cycle, which would indicate the need for a more holistic consortia of biological control agents. Further, some bacterial strains are known to impact on multiple targets, such as both fungi and RKN (Adam et al., 2014). As we did not observe this in the current study, we hypothesize that the fungal antagonists in our study would affect RKN with alternative mode of action than with nVOC.

## Conclusion

Our results indicate that VOC and plant-associated microbial diversity offers promise for RKN-defense management. Based on our data, we suggest three methods for RKN-control: (i) application of a consortia containing bacterial, fungi and nematode antagonists; (ii) application of sulfur-containing fertilizers to enhance sulfur-containing VOC in the rhizosphere for J2-reduction; (iii) screening and application of Rhizobiaceae-strains that produce nematicidal metabolites to take advantage of their increased abundance in galls. The combination of different management methods can lead to synergistic beneficial effects in tropical climates (Viaene et al., 2013). Implementing environmentally sensitive biocontrol strategies in agricultural programs, especially on smallholder farms, is an alternative to the harmful and often unspecific toxic biocides, toward preserving the stability and diversity of macro- and microhabitats. It would also help alleviate agriculture-related health issues, hunger and social conflicts while simultaneously providing economic and nutritional needs of the local people.

## Data Availability

The datasets generated and/or analyzed during the current study are available in the European Nucleotide Archive (ENA) under project no. PRJEB28436 under the accession numbers ERS2856266–ERS2856324. Reference sequences of *Meloidogyne incognita* (KJ476151.1), *M. javanica* (KP202352.1), *M. arenaria* (KP202350.1), *M. ethiopica* (KU372360), and *M. chitwoodi* (KJ476150.1) are publicly available at the NCBI database (https://www.ncbi.nlm.nih.gov/) under the corresponding accession numbers.

## Author Contributions

DC, GB, and AW designed the study. AW, JT, DC, RG, and GB performed the sample process. AW and JT analyzed the data. AW and GB wrote the manuscript. All authors improved and approved the final manuscript.

## Conflict of Interest Statement

The authors declare that the research was conducted in the absence of any commercial or financial relationships that could be construed as a potential conflict of interest.

## Footnotes

1 http://www.eurofinsgenomics.eu/

2 http://bioinformatics.psb.ugent.be/webtools/Venn/

3 https://view.qiime2.org
