# Supplementary material for: Novel Strategies for Soil-Borne Diseases: Exploiting the Microbiome and Volatile-Based Mechanisms Toward Controlling Meloidogyne-Based Disease Complexes
Source: Front Microbiol. 2019 Jun 7;10:1296. doi: 10.3389/fmicb.2019.01296 (PMC6568234; doi:10.3389/fmicb.2019.01296)
Supplement: Supplementary file 1 [file Data_Sheet_1.pdf]

Supplementary Material

1 Additional Methods.

Detailed description of nematode identification approaches, DNA preparation, PCRs, nematode breeding, dual cultures and single volatile organic compound tests.

1.1 Identification of RKN species:

Randomly selected adult females with dauer eggs were dissected from infected tomato roots with a sterile dissection needle; eggs were removed and females were transferred to 30 µl worm lysis buffer (WLB, after Castagnone-Sereno et al., 1995) on a microscope slide. Females were crushed using a needle, body content expelled and the *cuticulae* transferred to a microscope slide with tap water. Body contents with WLB were transferred to sterile 0.5 ml PCR tubes with 30 µl WLB, DNA extraction and PCR carried out following the method of Adam and colleagues (Adam et al., 2007). Gel electrophoresis was conducted in 0.5x TBE 0.8% agarose for 1 h at 100 V. A PCR with the primer pairs NAD5F2/NAD5R1 (Janssen et al., 2016) for NAD dehydrogenase subunit 5 sequences was used. Samples were purified using GENECLEAN Turbo™ Kit (MPBio) and DNA concentrations estimated with NanoDrop 2000 (Thermo Scientific, Wilmington, DE, USA). Sequencing was carried out by LGC Genomics GmbH (Berlin, GER). Sequences were checked for quality using Sequence Scanner 2.0 (Applied Biosystems), aligned using ClustalW implemented in MEGA 6 (Tamura et al., 2013) and compared to existing sequences using megablastn (https://blast.ncbi.nlm.nih.gov/Blast.cgi). Reference sequences from the NCBI database for several *Meloidogyne* species were added to the alignment and checked for species-specific SNP. According to observed SNP and data from Janssen and colleagues (Janssen et al., 2016), SCAR primer amplifications and perineal pattern morphology, the nematodes were assigned to a species.

1.2 Bacterial isolates and amplicon samples

A 5 g of soil was vortexed in 10 ml sterile 0.9% NaCl. 2*2 ml was transferred to 2*2 ml Eppendorf tubes. Samples were centrifuged for 20 min at 17,500 x g and 4°C. Supernatant was removed; remaining pellets stored at -20°C for amplicon analysis. 5 g of roots with adhering soil were bagmixed in sterile 50 ml 0.9% NaCl. 2*2 ml of the solution was transferred to Eppendorf tubes. Roots were surface-sterilized in 4% NaOCl for 3 min, washed 4 times with sterile distilled H_2O. Roots with root galling index (Coyne et al., 2007) (RGI) = 2 - 4.5 were seperated to gall infected (rhizoendosphere *Meloidogyne*-diseased, RE-D) and non-infected roots (rhizoendosphere healthy, RE-H). Root parts were weighed, placed into plastic bags with 5 ml sterile 0.9% NaCl and crushed with a mortar. 2x2ml resulting liquid was transferred to 2x2ml Eppendorf tubes, centrifuged for 20 min at 17,500 x g and 4°C. Supernatant was removed; remaining pellets stored at -20°C until further use.

Suspensions of root samples (rhizosphere, RE-H, RE-D) were diluted 10^{-1}, 10^{-2} and 10^{-3}. 100 µl of the dilutions plated on R2A plates with sterile glass beads and incubated at room temperature for two days.

Mean value for CFU/g root for every RGI rank was calculated. Semi-randomly selected, well-defined CFUs differing in shape, size and/or color were transferred to nutrient broth agar (NA) plates, resulting in 260 bacterial isolates. CFUs were incubated at 30°C for one day and stored at 4°C.

1.3 DNA preparation for amplicon analysis:

Extraction of DNA pellets was conducted with "FastDNA Spin Kit for soil" (MP Biomedical, Eschwege, GER) following the manufacturer's instruction with modifications to maximize DNA output. Soil and rhizosphere samples were cleaned using GENECLEAN Turbo™ Kit (MP Biomedicals, Eschwege, GER) following the manufacturer's instructions for genomic DNA. 16S rRNA amplifications were conducted in 3x30 µl reactions with Illumina barcode universal bacterial primer set 515f-806r (Caporaso et al., 2011) and PNA Mix (Lundberg et al., 2013) to remove plastid DNA. 2 µl template DNA, 1.2 µl of each primer, 6 µml Taq-&GO™ Mastermix 5xC (MP Biomedicals, Eschwege, GER) and 0.45 activated (heated 5min @ 55°C) PNA Mix was used. For PCR amplification conditions see Supplementary table S5**.** Gel electrophoresis was conducted in 1 x TAE buffer with 0.8% agarose using 100 V for 60 min. Samples with poor amplification bands were repeated another three times with 3µl template. For two samples (T16Gh, T17GH) the PCR was repeated with Q5®High-Fidelity DNA Polymerase (New England Biolabs, Frankfurt a. M., Germany) and 4 µl template. PCR products were purified using 'Wizard® SV Gel and PCR Clean-Up System' (Promega, Mannheim, Germany). DNA solutions were pooled to equimolarity, sequencing was conducted by Eurofins MWG Operon (Ebersberg, GER;http://www.eurofinsgenomics.eu/) with an Illumina HiSeq2500system.

1.4 Rearing of root-knot nematodes:

Four infected roots per sampling site were used for rearing. Tomato seedlings in the fourth to fifth true leaf stadium were grown in an autoclaved 1:1 (volume) mix of garden soil and sand. Two infected root sections (2-3 cm) were placed into the soil at 1 cm depth. Infected tomatoes were fertilized with long-term NPK fertilizer (COMPO Tomaten Langzeit-Dünger, COMPO GmbH & CO. KG, Germany) and grown for 50 days in a 16:8 h photoperiod at 25 ± 2°C. New tomato seedlings were infected with extracted second-stage juveniles (J2).

1.5 Bacterial antagonistic activity against fungal pathogens:

All bacterial isolates were tested for antagonistic activity against fungal pathogens *Botrytis cinerea*, *Fusarium oxysporum*, *Fusarium verticilloides*, *Sclerotium rolfsii* and *Verticillium dahliae* (all strains provided by Institute of Environmental Biotechnology Graz). Fungal pathogens were grown on potato dextrose agar (PDA) at room temperature for one week. For dual cultures with *B. cinerea*, *F. oxysporum*, *F. verticilloides* and *S. rolfsii*, bacterial isolates were streaked on Waksman agar plates (WA) in lines, four per plate with a gap in the center. Pieces of agar with hyphae were placed in the center. Plates were incubated at room temperature and controlled after 5 days. For *V. dahliae*, the method of Berg and colleagues (Berg et al., 1996) was applied. All tests were undertaken in triplicate. Antifungal activity was categorized according to: 0 (fungi overgrow bacterial colony), +1 (hyphae reach bacteria, but do not overgrow), +2 (lateral inhibition zone < 0.5 cm) and +3 (lateral inhibition zone > 0.5 cm). Mean category of all replicates was calculated. Bacteria showing strong antifungal effect (category +3) were compared using VENN (http://bioinformatics.psb.ugent.be/webtools/Venn/). DNA of strains with mean antifungal activity of +3 against at least four tested pathogens was isolated by transferring colonies to ribolysing tubes containing sterile glass beads and 400 µl distilled H_2O.

Samples were ribolysed two times for 30 s at 5.0 m/s and centrifuged at 17,500 x g for 5 min. Supernatant was used for fingerprint BOX PCR (Rademaker and de Bruin, 1997) and 16S rDNA sequencing. BOX-PCR was conducted in 25 µl reactions containing 2.5 µl 10 µM BOX A1 primer (Rademaker and de Bruin, 1997), 5 µl Taq-&GO™ Mastermix 5xC and 1 µl template. For bacterial isolates with different amplification patterns, 16S rDNA was amplified using the universal bacterial primer set 27f/1492r in 30 µl reactions, containing 2 µl template, 1 µl of each primer (10 µM) and 6 µl Taq-&GO™ Mastermix 5xC.

1.6 SPME GC-MS of nVOC-producing bacteria:

We used a variation of the method from Verginer and colleagues (Verginer et al., 2010): nVOC-producing strains were streaked on 8 mL NA slope agar (1.5%) within 20 mL headspace vials (75.5 x 22.5 mm; Chromtech, Idstein, Germany). Vials were closed with cotton plugs and covered with aluminum foil. After 24 h incubation at 30°C, vials were sealed with crimp seals and incubated for an additional 2 h. Solid phase micro extraction (SPME) was performed with an automated sampler and a 50/30 µm Divinylbenzen/CarboxenTM/Polydimethylsiloxane (PDMS) 2 cm Stableflex/SSfiber (Supelco, Bellefonte, PA, USA). Volatile compounds were extracted for 30 min at 30°C, desorption time in the injector was 36 min. Compound separation and detection was performed on a system combining a GC7890A with a quadrupole MS5975C (Agilent Technologies, Waldbronn, Germany). Samples were analyzed by a (5%-phenyl)methylpolysiloxane column, 30 m × 0.25 mm i.d., 0.25 µm film thickness (HP-5MS; Agilent Technologies, Waldbronn, Germany), followed by electron ionization (EI; 70 eV) and detection (mass range 25–350). Inlet temperature was adjusted to 250°C. The following temperature program was applied: 40°C for 2 min, raising to 110°C at a rate of 5°C/min, then to 280°C at 10°C /min and finally maintained at 280°C for 3 min. Helium flow rate was set to 1.2 mL/min. Obtained spectra were compared with NIST Mass Spectral Database 08 entries. Specific compounds were identified based on values for NIST database matches and on non-isothermal Kovats retention indices calculated after Van den Dool and Kratz (1963).

1.7 Testing nematicidal effect of single VOC:

A total of 9 single compounds (purity > 98%) partially found in GC-MS samples were tested against J2 in a chambered Petri dish, namely decene (10en), undecene (11en), undecan-2-on (11on), dodecene (12en), 2-methoxy-3-methyl pyrazine (2M3MP), 2,5-dimethyl pyrazine (25DP), 5-isobutyl-2,3-dimethyl pyrazine (5I23DP), 2-ethyl-3-methyl pyrazine (2E3MP) and 2-isobutyl-3-methoxy pyrazine (2I3MP) (all Sigma-Aldrich, Darmstadt, Germany). On one side of the Petri dish, 500 µl of *M. javanica*-suspension were placed on 8 ml of 2%-tab water agar. On the other side, single compounds were pipetted in three different volumes (1 µl, 5 µl, 20 µl) on a microscopic slide to prevent interactions of compounds with petri dish material. Tests were incubated at room temperature for 24 h, then proportion of dead J2 were counted. 20 µl distilled water was used as blank. Tests were repeated three times and the blank-corrected mortality rate was calculated for all nine compounds.

2 Additional Results

Detailed information regarding the number of isolated bacteria and abundant nematode species.

2.1 Cultivable bacteria from tomato root endosphere:

The number of cultivable bacterial strains extracted from endosphere was highest in plants with moderately infected roots (RGI = 2.5, RE-H: n = $5*10^5$ CFU/g root; RE-D: n = $2.7*10^5$ CFU/g root) and lowest in severely infected (RGI = 4, RE-H: n =744 CFU/g root; RE-D: n = 195 CFU/g root). More bacteria could be cultured from lethal infected plants (RGI = 5, RE-D: n = $8.4*10^3$ CFU/g root) than from healthy plants (RGI = 1, RE-H: n = $2*10^3$ CFU/g root). Average number of CFU/g root was higher in healthy roots (RE-H) at all infection categories except for RGI = 3.

2.2 Abundant nematode species:

Due to the perineal pattern morphology, the extracted adult females could be assigned to either *M. incognita s. str.*, which showed no lateral lines and a relatively high, square-shaped dorsal arch, *M. javanica* having the typical rounded pattern with distinct lateral lines or members of the *M. incognita*-group (MIG). There was a variance within the patterns that may indicate more than these two species. Those specimens were assigned to *M. incognita. s. lat.*

The molecular key diagnosis from Adam and colleagues (Adam et al., 2007) did not give clear results, only 7 of 30 nematodes could be assigned to one species due to SCAR-primer. Other samples did not reveal amplification bands in electrophoresis gels. No amplifications of *M. arenaria*-specific primers could be detected. The amplified samples could be assigned to either *M. javanica* or *M. incognita*.

When comparing nematode sample sequences of NAD5 with the reference genomes of different *Meloidogyne* species, some sequences showed several positions with poor quality and/or SNP. When using a neighbor-joining tree, eight samples did not cluster with the reference genome sequences (Supplementary Figure S3). Therefore, species-specific polymorphisms within the reference genomes were used for assigning a sample to a species.

No samples could be assigned to *M. arenaria*. 10 specimens of *M. incognita* and two MIG specimens were found on Luwero (site 1). Three specimens of *M. incognita*, six of *M. javanica* and five of MIG were identified from Namulonge (site2).

3 Supplementary Figures

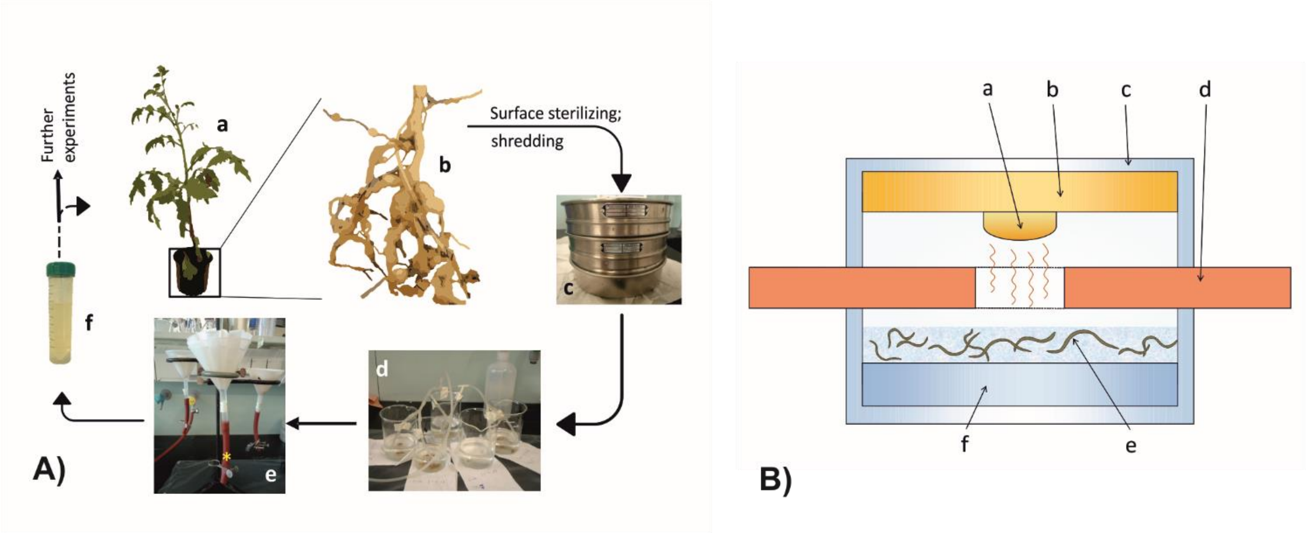

Supplementary Figure S1. A: Workflow of second-stage juveniles (J2) extraction from diseased tomato roots. **a**: diseased tomato plant; **b**: roots with visible galls is surface sterilized and shredded; **c**: root suspension is washed through nested 100 µm and 25 µm sieves, eggs are caught on the 25 µm sieve; **d**: egg suspension is aerated for 10 days; **e**: Baermann funnel. Hatched J2 actively migrate through filter paper to the lower part of the rubber hose (asterisk); **f**: J2 suspension for experiments or reinfection of new tomato seedlings. **B:** Experimental setup of modified Two-clamp volatile assay (TCVA) for nematicidal volatile organic compound (nVOC)-producing bacteria**. a**: Volatile-producing bacterial strains; **b**: nutrient broth agar; **c**: 12-well plates; **d**: sterile silicone foil septum with perforation; **e:** J2 suspension of *Meloidogyne incognita*; **f**: 2% H_2O agar.

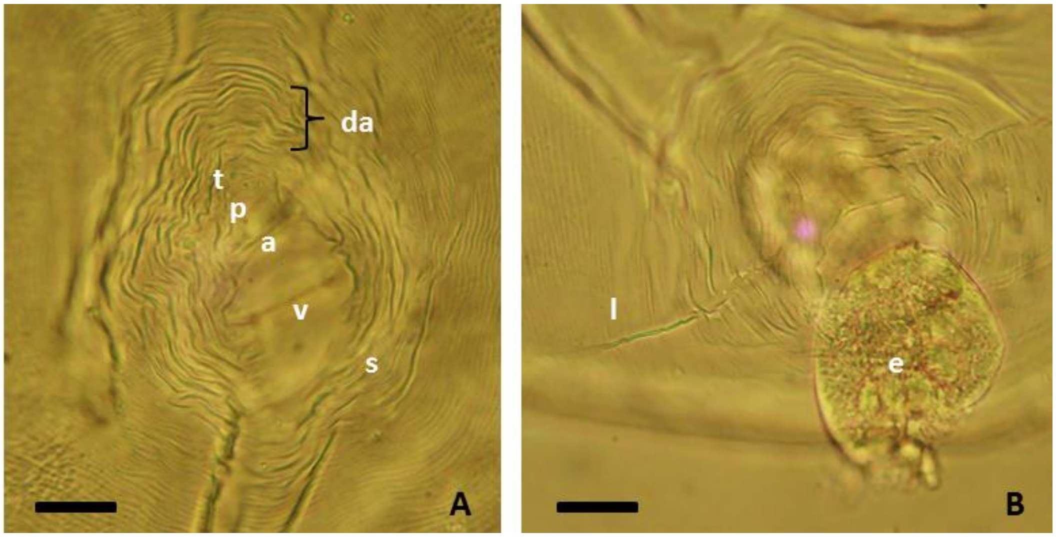

Supplementary Figure S2. Perineal Pattern of *Meloidogyne incognita* (A) and egg-laying *M. javanica* (B). Typical are the high dorsal arch for *M. incognita* and the lateral lines in *M. javanica*. **a**: anus; **da**: dorsal arch; **e**: egg; **l**: lateral lines; **p**: punctuations; **s**: striae; **t**: tail terminus; **v**: vulva.

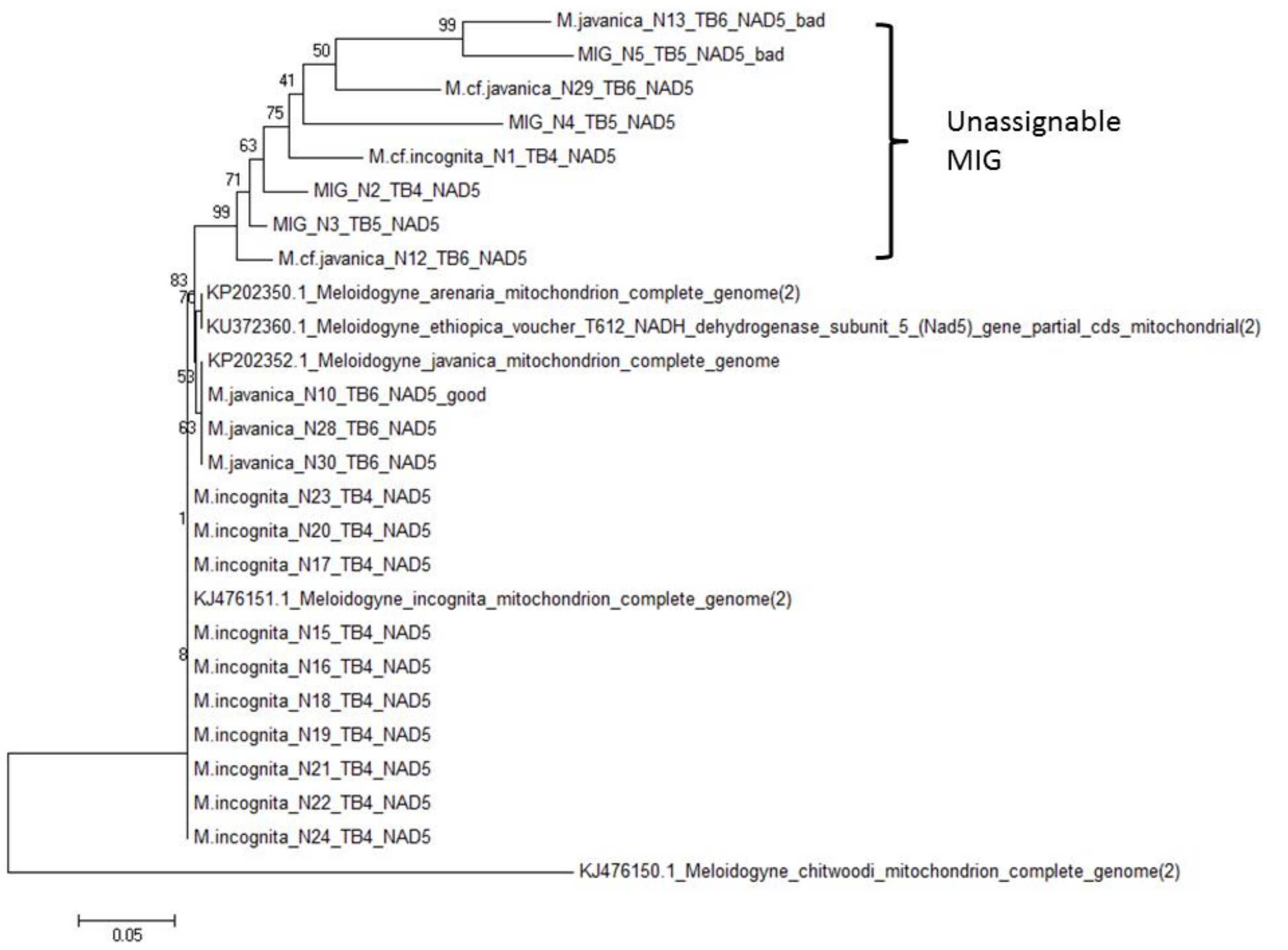

Supplementary Figure S3. Neighbour-joining tree of NAD5 of extracted nematodes. Eight samples were unassignable on overall sequences due to poor quality sequences and thus assigned using SNPs and perineal pattern morphology.

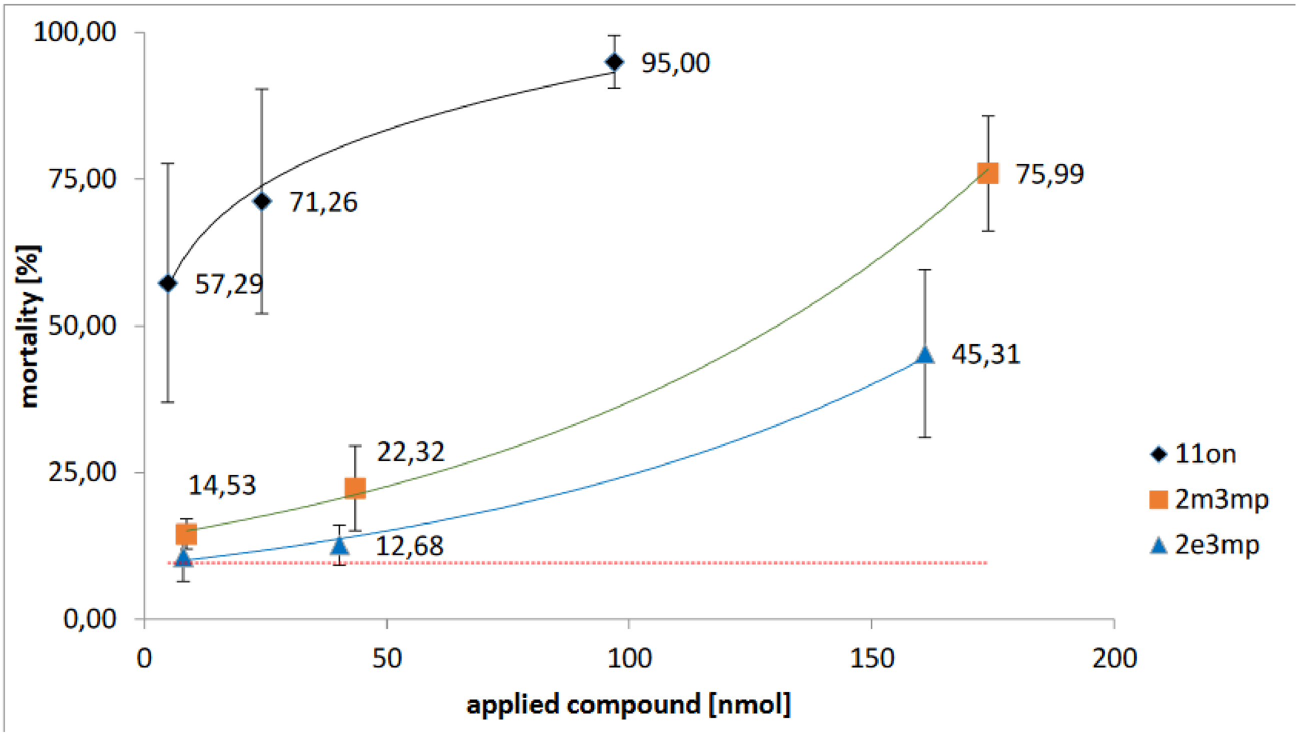

Supplementary Figure S4. Single compounds that revealed nematicidal activity after 24 h of incubation. 11on: undecan-2-on; 2m3mp: 2-methoxy-3-methyl pyrazine; 2e3mp: 2-ethyl-3-methyl pyrazine. Red line represents blank mortality. Compounds with no nematicidal activity are not shown.

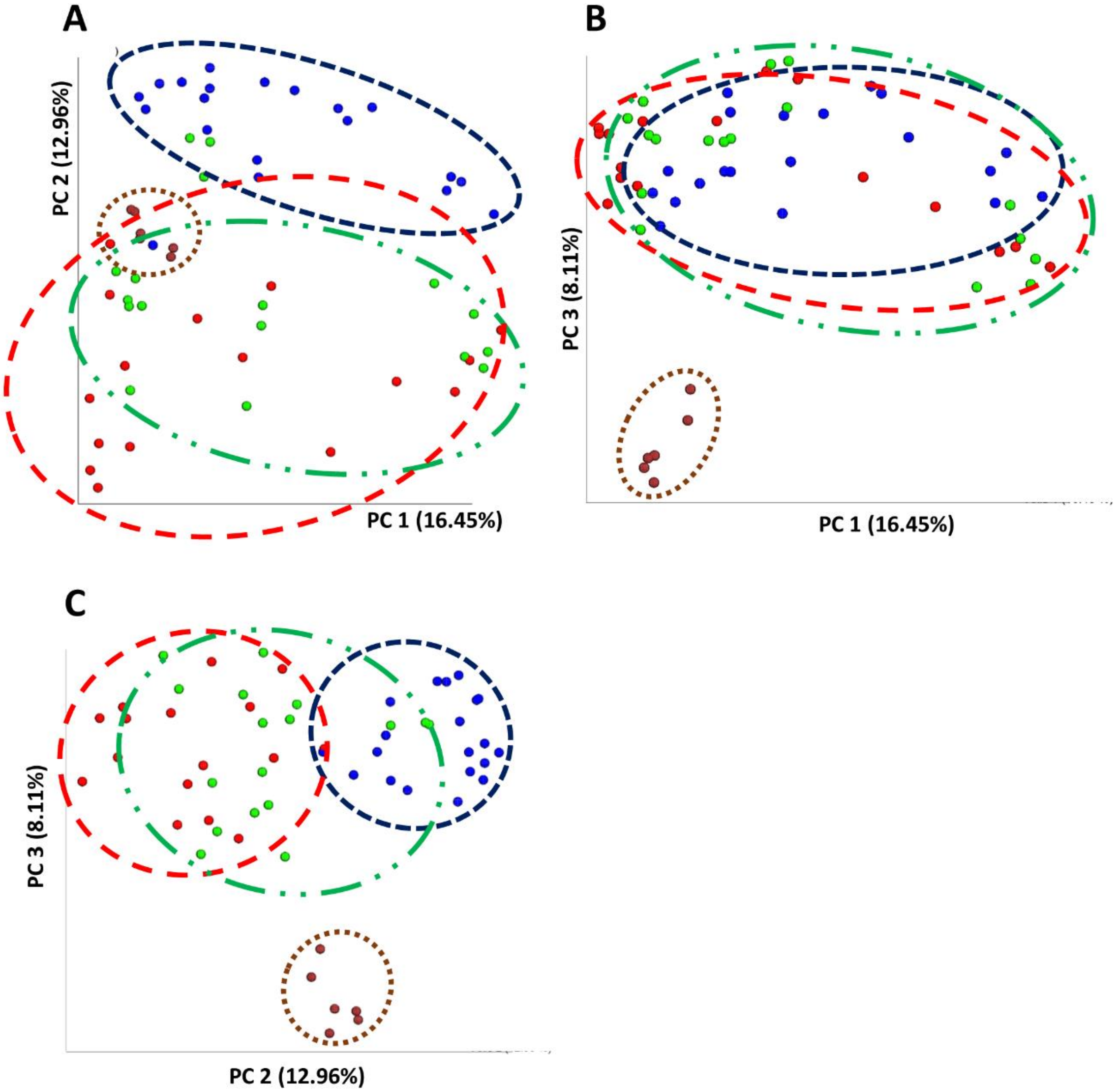

Supplementary Figure S5. Two-dimensional PCoA-plots of Bray-Curtis dissimilarity of 16SrRNA amplicon samples. Samples do not cluster according to habitat with axis 1 (A, B), but with axis 2 and 3 (C). Brown: soil; blue: rhizosphere; green: healthy root endosphere (RE-H); red: diseased root endosphere (RE-D).

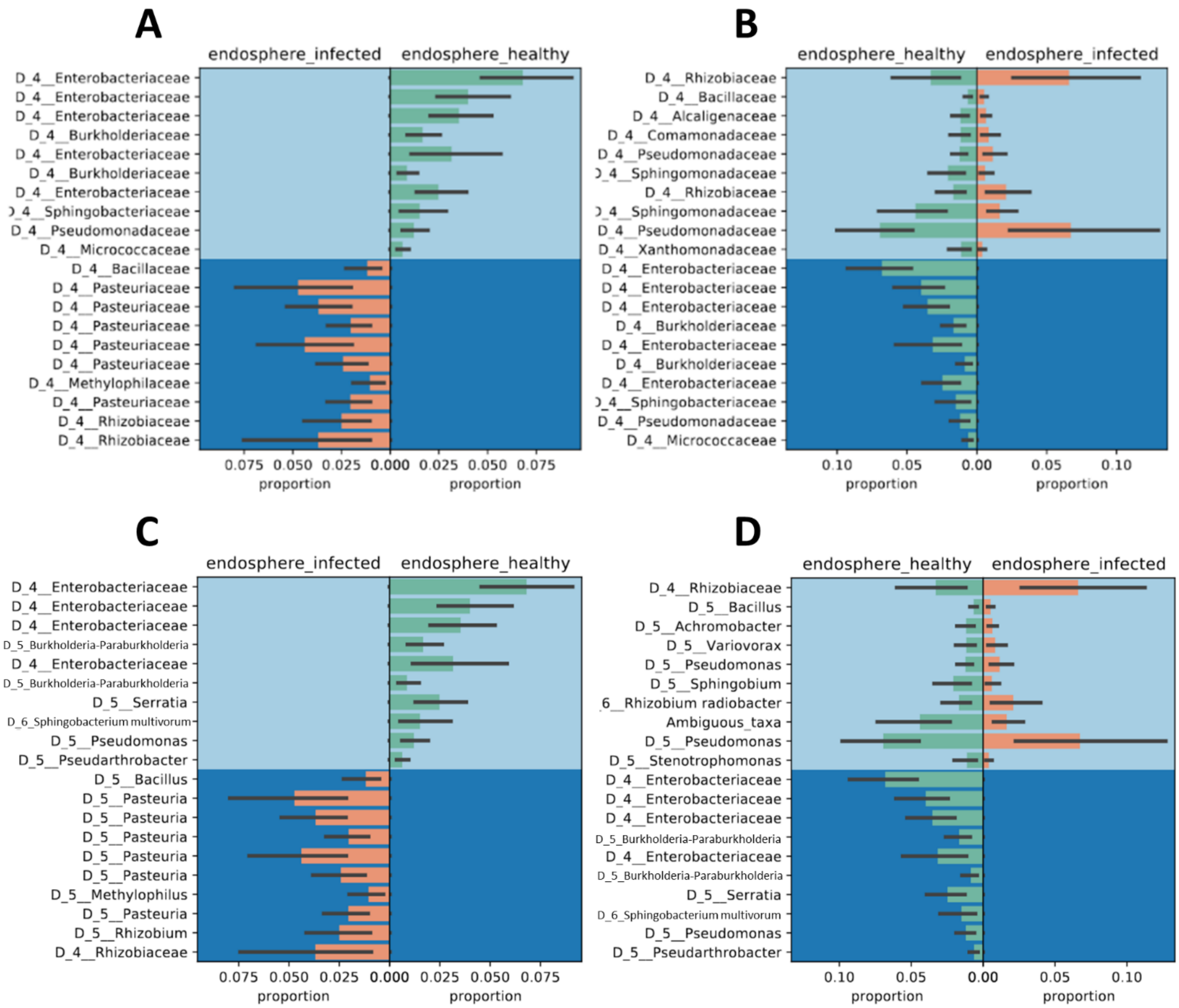

Supplementary Figure S6. Proportion plot of balances on level 4 (A, B) and level 6 (C, D) constructed with differential abundance analysis using GNEISS implemented in QIIME2 (v. 12.2017). Plot of the two linear regressions y0 (A, C) and y2 (B, D). Most influencing taxa are shown. On level 6, highest possible identification level did partially not exceed family, repetitions represent different, yet unidentifiable taxa. Taxa of enumerator light blue, taxa of denominator in dark blue.

4 Supplementary Tables

Supplementary Table S1. Identification references to compounds found in the volatilome of bacterial nematode antagonists using SPME GC-MS.

	RI measured[4]	RI NIST (mainlib)	References
1-nonene	889	892	(Flamini et al., 2003)
1-decene	989	993	(Flamini et al., 2003)
1-undecene	1091	1093	(Flamini et al., 2003)
E-1,4-undecadiene	1081	-	
E-3-undecene	1085	1085	(Junkes et al., 2003)
1-dodecene	1186	1193	(Flamini et al., 2003)
1-tridecene	1286	1293	(Flamini et al., 2003)
1,12-tridecadiene	1272	1279	(Sojáka et al., 2006)
Methanethiol	-	464	(Bonaïti et al., 2005)
Dimethyl sulfide	520	515	(Bonaïti et al., 2005)
Dimethyl disulfide	733	740	(Bonaïti et al., 2005)
Dimethyl trisulfide	959	972	(Jarunrattanasri et al., 2007)
S-methyl propanethioate	784	785	(Gros et al., 2011)
2,3-dimercaptopropan-1-ol	835	-	
2-me-2-methylthiobutan	836	847	(Liu et al., 2005)
Methyl thiolacetate	698	701	(Beaulieu and Grimm, 2001)
S-methyl 3-methyl-butanethioate	931	938	(Beaulieu and Grimm, 2001)
S-methyl ester octanethioic acid	1291	1293	(Gros et al., 2011)
CO_2	-	-	
Acetone	502	503	(Insausti et al., 2005)
2-butanone	601	601	(Insausti et al., 2005)
2-methyl butanal	656	659	(Jarunrattanasri et al., 2007)
3-methyl butanal	646	649	(Jarunrattanasri et al., 2007)
2-methyl butanol	728	736	(Pino et al., 2005)
3-methyl butanol	725	734	(Pino et al., 2005)
2,3-epoxybutan	510	-	
Benzaldehyde	951	958	(Kukic et al., 2006)
2-undecanone	1288	1291	(Vagionas et al., 2007)
3-methoxy-2,5-dimethyl pyrazine	1046	1054	(Jagella and Grosch, 1999)

Supplementary Table S2. Mass spectra of compounds identified in the volatilome of bacterial nematode antagonists.

compounds	fragmentation patterns m/z (relative intensity)
CO_2	44 (M+, 100), 40 (34), 45 (13)
Methanethiol	47 (100), 48 (M+, 82), 45 (66), 44 (22), 46 (15), 49 (6), 50 (4),
Acetone	43 (100), 44 (39), 58 (M+, 39), 45 (19), 47 (17), 48 (14), 40 (9), 42 (7), 39 (5), 41 (3)
2,3-epoxybutan	43 (100), 44 (79), 58 (36), 47 (24), 48 (20), 45 (16), 68 (15), 40 (15), 39 (13), 42 (9)
Dimethyl sulfide	44 (100), 47 (40), 62 (M+, 33), 43 (26), 45 (25), 40 (15), 48 (15), 46 (13), 61 (11), 58 (11)
2-butanone	43 (100), 72 (M+, 33), 44 (29), 57 (12), 40 (8), 42 (7), 41 (3), 39 (3), 45 (2), 47 (2)
3-methyl butanal	44 (100), 41 (86), 43 (70), 58 (63), 39 (38), 71 (29), 57 (25), 42 (20), 45 (12), 40 (8)
2-methyl butanal	41 (100), 57 (88), 58 (65), 56 (53), 43 (40), 39 (30), 42 (18), 44 (18), 55 (18), 40 (11)
Methyl thiolacetate	43 (100), 90 (M+, 65), 45 (17), 47 (14), 44 (9), 42 (7), 46 (6), 48 (6), 75 (5), 92 (3)
3-methyl butanol	55 (100), 70 (68), 43 (65), 42 (61), 41 (56), 44 (45), 39 (24), 57 (24), 45 (18), 40 (13)
2-methyl butanol	57 (100), 44 (98), 56 (89), 41 (85), 70 (61), 55 (53), 43 (46), 42 (30), 39 (26), 40 (22)
Dimethyl disulfide	94 (M+, 100), 79 (52), 45 (37), 46 (18), 47 (14), 64 (11), 61 (10), 96 (10), 48 (6), 81 (5)
S-methyl propanethioate	57 (100), 104 (M+, 54), 45 (27), 47 (23), 94 (19), 75 (17), 48 (13), 46 (10), 79 (10), 61 (95)
2,3-dimercaptopropan-1-ol	59 (100), 106 (65), 58 (23), 45 (14), 57 (8), 91 (7), 47 (7), 61 (6), 108 (6), 44 (5)
2-me-2-methylthiobutan	43 (100), 71 (60), 41 (40), 118 (M+, 23), 48 (20), 75 (19), 47 (18), 45 (16), 39 (16), 44 (11)
1-nonene	43 (100), 90 (M+, 59), 45 (17), 44 (17), 47 (14), 42 (7), 48 (6), 46 (6), 75 (4), 92 (3)
Methyl (Z)-N-hydroxybenzene-carboximidate	133 (100), 151 (M+, 69), 135 (23), 77 (14), 134 (13), 68 (10), 152 (9), 45 (6), 75 (6), 153 (6)
S-methyl 3-methyl-butanethioate	57 (100), 85 (75), 41 (47), 75 (20), 43 (19), 39 (16), 47 (14), 45 (12), 42 (8), 56 (8)
Benzaldehyde	106 (M+, 100), 105 (98), 77 (95), 51 (35), 50 (21), 78 (18), 74 (10), 52 (9), 107 (8), 76 (5)
Dimethyl trisulfide	126 (M+, 100), 79 (46), 45 (33), 47 (20), 64 (19), 111 (16), 80 (14), 128 (13), 46 (12), 78 (7)
1-decene	55 (100), 56 (99), 41 (97), 70 (88), 69 (72), 43 (68), 57 (68), 83 (42), 39 (39), 42 (33)
3-methoxy-2,5-dimethyl pyrazine	138 (M+, 100), 109 (49), 137 (44), 120 (32), 107 (28), 82 (28), 54 (28), 42 (249), 95 (17), 108 (16)
E-1,4-undecadiene	67 (100), 54 (89), 81 (74), 68 (67), 41 (66), 55 (64), 69 (50), 82 (48), 79 (46), 43 (37)
E-3-undecene	55 (100), 41 (92), 70 (85), 56 (83), 43 (79), 69 (78), 83 (61), 57 (55), 84 (40), 97 (37)
1-undecene	55 (100), 70 (95), 69 (88), 41 (86), 56 (83), 83 (73), 43 (68), 57 (56), 84 (49), 97 (49)

1-dodecene	55 (100), 41 (88), 56 (82), 43 (81), 69 (79), 70 (75), 83 (65), 57 (58), 97 (50), 84 (40)
1,12-tridecadiene	67 (100), 81 (82), 55 (76), 41 (65), 54 (60), 82 (57), 68 (46), 96 (43), 95 (38), 69 (38)
1-tridecene	55 (100), 41 (91), 43 (89), 69 (84), 83 (77), 70 (74), 56 (73), 57 (70), 97 (59), 84 (38)
2-undecanone	58 (100), 43 (71), 71 (35), 59 (30), 41 (18), 55 (11), 57 (11), 85 (10), 39 (6), 42 (6)
S-methyl ester octanethioic acid	57 (100), 127 (62), 43 (50), 58 (32), 41 (31), 55 (24), 71 (12), 159 (12), 75 (10), 39 (10)
unknown substance (Rt=1.581)	44 (100), 47 (82), 48 (69), 45 (48), 40 (22), 46 (12), 49 (5), 50 (4)
unknown substance (Rt=12.277)	57 (100), 43 (99), 71 (95), 85 (68), 41 (42), 55 (25), 70 (25), 84 (22), 69 (20), 56 (19)

Supplementary Table S3. Percental relative abundance shift of bacterial orders. Δ(abundance) refers to the assumption of abundance to be 100% in the ‘previous’ habitat (rhizosphere → RE-H → RE-D). Red arrow: < -50%; green arrow: > +50%; yellow arrow: ± 50% range. *Enterobacteriales* and *Bacillales* increase in the endosphere, but in diseased roots *Enterobacteriales* become less abundant. *Rhizobiales* abundance rises in diseased tomato roots. Percental relative abundance shifts are higher in low-abundant orders.

rhizosphere		RE-H		RE-D	
**order**	**rA[%]**	**rA[%]**	**Δ(abundance) [%]**	**rA[%]**	**Δ(abundance) [%]**
Psudomonadales	25.74	9.85	-61.75	8.67	-11.92
Enterobacteriales	17.35	32.29	+86.19	24.7	-23.52
Burkholderiales	10.3	12.56	+21.95	10.03	-20.14
Sphingomonadales	9.86	4.27	-56.7	2.21	-48.1
Rhizobiales	7.5	6.29	-16.08	12.43	+97.49
Sphingobacteriales	6.74	3.54	-47.5	2.18	-38.53
Flavobacteriales	3.47	1.95	-43.93	1.31	-32.71
Xanthomonadales	3.34	3.57	+6.59	1.92	-46.05
Bacillales	3.06	11.45	+274.4	27.65	+141.36
Caulobacterales	1.21	1.34	+10.4	0.65	-52.41
Cytophagales	1.19	0.53	-55.58	0.42	-19.87
Methylophilales	1.18	1.76	+48.95	0.88	-50.17
Micrococcales	1.13	1.25	+10.94	0.92	-26.04
orders <1% rA	7.93	9.89	+24.72	8.91	-9.97

Supplementary Table S4. List of all primers used in this study with references.

Primer Name	Sequence (5'-3')	Specificity	Reference
Nem_194	TTAACTTGCCAGATCGGACG	5S-18S ribosome region	(Blok et al., 1997)
Nem_195	TCTAATGAGCCGTACGC		
Nem_Far	TCGGCGATAGAGGTAAATGAC	*M. arenaria*-specific SCAR	(Zijlstra et al., 2000)
Nem_Rar	TCGGCGATAGACACTACAAACT		
Nem_Fjav	GGTGCGCGATTGAACTGAGC	*M. javanica*-specific SCAR	(Zijlstra et al., 2000)
Nem_Rjav	CAGGCCCTTCAGTGGAACTATAC		
Nem_MI-F	GTGAGGATTCAGCTCCCCAG	*M. incognita*-specific SCAR	(Meng et al., 2004)
Nem_MI-R	ACGAGGAACATACTTCTCCGTCC		
Nem_NAD5F2	TATTTTTTGTTTGAGATATATTAG	NADH dehydrogenase subunit 5	(Janssen et al., 2016)
Nem_NAD5R1	CGTGAATCTTGATTTTCCATTTTT		
Nem_D2A	ACAAGTACCGTGAGGGAAAGTT	D2-D3 expansion of 28S rDNA	(Baldwin et al., 1997)
Nem_D3B	TCGGAAGGAACCAGCTACTA		
515f	GTGYCAGCMGCCGCGGTAA	16S rDNA	
806r	GGACTACHVGGGTWTCTAAT		
27f	AGAGTTTGATCMTGGCTCAG	16S rDNA	
1492r	GGTTACCTTGTTACGACTT		
BOX A1R	CTACGGCAAGGCGACGCTGACG	Repetitive sequences	(Rademaker and de Bruin, 1997)

Supplementary Table S5. List of PCR conditions for all primers used in this study.

Primer	Melting 1	Melting 2	PNA annealing	Primer annealing	Elongation	Repeats (grey)	Elongation	cooling
**515f/806r pNA Amplicon PCR**	5min @ 96°C	60s @ 96°C	5s @ 75°C	60s @ 54°C	60s @ 74°C	30x	10min @ 74°C	hold @ 4°C
**27f/1492r**	5min @ 95°C	30s @ 95°C		30s @ 57°C	90s @ 72°C	30x	5min @ 72°C	hold @ 15°C
**BOX PCR**	6min @ 95°C	60s @ 95°C	-	60s @ 53°C	8m @ 65°C	35x	16min @ 65°C	hold @ 10°C
**NAD5_F2/R1**	2min @ 94°C	60s @ 94°C	-	60s @ 45°C	90s @ 72°C	40x	10min @ 72°C	hold @ 4°C
**D2A/D3B**	4min @ 94°C	60s @ 94°C	-	90s @ 55°C	120s @ 72°C	35x	10min @ 72°C	hold @ 4°C
**Nem_194/195**	2min @ 94°C	30s @ 94°C	-	30s @ 50°C	90s @ 72°C	45x	7min @ 72°C	hold @ 4°C
**Nem_Far/Rar**	2min @ 94°C	30s @ 94°C	-	30s @ 61°C	60s @ 72°C	45x	7min @ 72°C	hold @ 4°C
**Nem_Fjav/Rjav**	2min @ 94°C	30s @ 94°C	-	30s @ 64°C	60s @ 72°C	45x	7min @ 72°C	hold @ 4°C
**Nem_MI-F/MI-R**	2min @ 94°C	30s @ 94°C	-	30s @ 62°C	60s @ 72°C	45x	7min @ 72°C	hold @ 4°C

5 References

Adam, M. A. M., Phillips, M. S., and Blok, V. C. (2007). Molecular diagnostic key for identification of single juveniles of seven common and economically important species of root-knot nematode (Meloidogyne spp.). *Plant Pathol.* 56, 190–197. doi:10.1111/j.1365-3059.2006.01455.x.

Baldwin, J. G., Frisse, L. M., Vida, J. T., Eddleman, C. D., and Thomas, W. K. (1997). An evolutionary framework for the study of developmental evolution in a set of nematodes related to Caenorhabditis elegans. *Mol. Phylogenet. Evol.* 8, 249–59. doi:10.1006/mpev.1997.0433.

Beaulieu, J. C., and Grimm, C. C. (2001). Identification of Volatile Compounds in Cantaloupe at Various Developmental Stages Using Solid Phase Microextraction. *J. Agric. Food Chem.* 49, 1345–1352. doi:10.1021/jf0005768.

Berg, G., Marten, P., and Ballin, G. (1996). Stenotrophomonas maltophilia in the rhizosphere of oilseed rape - Occurrence, characterization and interaction with phytopathogenic fungi. *Microbiol. Res.* 151, 19–27. doi:10.1016/S0944-5013(96)80051-6.

Blok, V. C., Phillips, M. S., and Fargette, M. (1997). Comparison of Sequences from the Ribosomal DNA Intergenic Region of Meloidogyne mayaguensis and Other Major Tropical Root-Knot Nematodes. *J. Nematol.* 29, 16–22.

Bonaïti, C., Irlinger, F., Spinnler, H. E., and Engel, E. (2005). An Iterative Sensory Procedure to Select Odor-Active Associations in Complex Consortia of Microorganisms: Application to the Construction of a Cheese Model. *J. Dairy Sci.* 88, 1671–1684. doi:https://doi.org/10.3168/jds.S0022-0302(05)72839-3.

Caporaso, J. G., Lauber, C. L., Walters, W. A., Berg-Lyons, D., Lozupone, C. A., Turnbaugh, P. J., et al. (2011). Global patterns of 16S rRNA diversity at a depth of millions of sequences per sample. *Proc. Natl. Acad. Sci.* 108, 4516 LP-4522. Available at: http://www.pnas.org/content/108/Supplement_1/4516.abstract.

Castagnone-Sereno, P., Esparrago, G., Abad, P., Leroy, F., and Bongiovanni, M. (1995). Satellite DNA as a target for PCR-specific detection of the plant-parasitic nematode Meloidogyne hapla. *Curr. Genet.* 28, 566–70. doi:10.1007/BF00518170.

Coyne, D. L., Nicol, J. M., and Claudius-Cole, B. (2007). *Practical plant nematology: A field and laboratory guide*. SP-IPM Secretariat, International Institute of Tropical Agriculture (IITA), Cotonou, Benin.

Flamini, G., Cioni, P. L., and Morelli, I. (2003). Differences in the fragrances of pollen, leaves, and floral parts of garland (Chrysanthemum coronarium) and composition of the essential oils from flowerheads and leaves. *J. Agric. Food Chem.* 51, 2267–2271. doi:10.1021/jf021050l.

Gros, J., Nizet, S., and Collin, S. (2011). Occurrence of Odorant Polyfunctional Thiols in the Super Alpha Tomahawk Hop Cultivar. Comparison with the Thiol-rich Nelson Sauvin Bitter Variety. *J. Agric. Food Chem.* 59, 8853–8865. doi:10.1021/jf201294e.

Insausti, K., Goñi, V., Petri, E., Gorraiz, C., and Beriain, M. J. (2005). Effect of weight at slaughter on the volatile compounds of cooked beef from Spanish cattle breeds. *Meat Sci.* 70, 83–90. doi:https://doi.org/10.1016/j.meatsci.2004.12.003.

Jagella, T., and Grosch, W. (1999). Flavour and off-flavour compounds of black and white pepper (Piper nigrum L.). *Eur. Food Res. Technol.* 209, 16–21. doi:10.1007/s002170050449.

Janssen, T., Karssen, G., Verhaeven, M., Coyne, D., and Bert, W. (2016). Mitochondrial coding genome analysis of tropical root-knot nematodes (Meloidogyne) supports haplotype based diagnostics and reveals evidence of recent reticulate evolution. *Sci. Rep.* 6, 1–13. doi:10.1038/srep22591.

Jarunrattanasri, A., Theerakulkait, C., and Cadwallader, K. R. (2007). Aroma Components of Acid-Hydrolyzed Vegetable Protein Made by Partial Hydrolysis of Rice Bran Protein. *J. Agric. Food Chem.* 55, 3044–3050. doi:10.1021/jf0631474.

Junkes, S. B., Amboni, R. D. M. C., Yunes, A. R., and Heinzen, V. E. F. (2003). Semiempirical Topological Index: A NovelMolecular Descriptor for Quantitative Structure–Retention Relationship Studies. *Internet Electron. J. Mol. Des.* 2, 33–49.

Kukic, J., Petrovic, S., Pavlovic, M., Couladis, M., Tzakou, O., and Niketic, M. (2006). Composition of essential oil of Stachys alpina L. ssp. dinarica Murb. *Flavour Fragr. J.* 21, 539–542. doi:10.1002/ffj.1684.

Liu, L., Cao, C.-Z., Xie, B., and Zou, L.-K. (2005). Research of QSSR on chromatography retention index of sulfides and mercaptans. *J. Hunan Univ. Sci. Technol.* 20, 74–80.

Lundberg, D. S., Yourstone, S., Mieczkowski, P., Jones, C. D., and Dangl, J. L. (2013). Practical innovations for high-throughput amplicon sequencing. *Nat. Methods* 10, 999. Available at:

http://dx.doi.org/10.1038/nmeth.2634.

Meng, Q. P., Long, H., and Xu, J. H. (2004). PCR assays for rapid and sensitive identification of three major root-knot nematodes, Meloidogyne incognita, M. javanica and M. arenaria. *Acta Phytopathol. Sin.* 34, 204–210.

Pino, J. A., Mesa, J., Muñoz, Y., Martí, M. P., and Marbot, R. (2005). Volatile Components from Mango (Mangifera indica L.) Cultivars. *J. Agric. Food Chem.* 53, 2213–2223. doi:10.1021/jf0402633.

Rademaker, J. L. W., and de Bruin, F. J. (1997). “Characterization and classification of microbes by rep-PCR genomic fingerprinting and computer assisted pattern analysis,” in *DNA markers: protocols, applications and overviews*, eds. G. Caetano-Anollés and P. M. Gresshoff (New York, N.Y.: J. Wiley and Sons), 151–171.

Sojáka, L., Kubineca, R., Jurdáková, H., Hájeková, E., and Bajus, M. (2006). Gc-Ms of Polyethylene and Polypropylene Thermal Cracking Products. *Pet. Coal* 48, 1–14.

Tamura, K., Stecher, G., Peterson, D., Filipski, A., and Kumar, S. (2013). MEGA6: Molecular evolutionary genetics analysis version 6.0. *Mol. Biol. Evol.* 30, 2725–2729. doi:10.1093/molbev/mst197.

Vagionas, K., Ngassapa, O., Runyoro, D., Graikou, K., Gortzi, O., and Chinou, I. (2007). Chemical analysis of edible aromatic plants growing in Tanzania. *Food Chem.* 105, 1711–1717. doi:https://doi.org/10.1016/j.foodchem.2007.05.029.

Van den Dool, H., and Kratz, P. D. (1963). A generalization of the retention index system including linear temperature programmed gas liquid partition chromatography. *J. Chromatogr.* 11, 463–71.

Verginer, M., Leitner, E., and Berg, G. (2010). Production of volatile metabolites by grape-associated microorganisms. *J. Agric. Food Chem.* 58, 8344–8350. doi:10.1021/jf100393w.

Zijlstra, C., Donkers-Venne, D. T. H. M., and Fargette, M. (2000). Identification of *Meloidogyne incognita*, *M. javanica* and *M. arenaria* using sequence characterised amplified region (SCAR) based PCR assays. *Nematology* 2, 847–853. doi:https://doi.org/10.1163/156854100750112798.
